# Neuronal polyunsaturated fatty acids are protective in ALS/FTD

**DOI:** 10.1038/s41593-025-01889-3

**Published:** 2025-02-25

**Authors:** Ashling Giblin, Alexander J. Cammack, Niek Blomberg, Sharifah Anoar, Alla Mikheenko, Mireia Carcolé, Magda L. Atilano, Alex Hull, Dunxin Shen, Xiaoya Wei, Rachel Coneys, Lele Zhou, Yassene Mohammed, Damien Olivier-Jimenez, Lian Y. Wang, Kerri J. Kinghorn, Teresa Niccoli, Alyssa N. Coyne, Rik van der Kant, Tammaryn Lashley, Martin Giera, Linda Partridge, Adrian M. Isaacs

**Affiliations:** 1 UK Dementia Research Institute, UCL, London, UK; 2 Institute of Healthy Ageing, UCL, London, UK; 3 Department of Neurodegenerative Disease, UCL Queen Square Institute of Neurology, London, UK; 4 Center for Proteomics & Metabolomics, Leiden University Medical Center, Leiden, The Netherlands; 5 Department of Neurology, Johns Hopkins University, Baltimore, MA USA; 6 Brain Science Institute, Johns Hopkins University, Baltimore, MA USA; 7 Alzheimer Center Amsterdam, Amsterdam University Medical Center, Amsterdam, The Netherlands; 8 Present Address: MRC Laboratory of Molecular Biology, Cambridge, UK

**Keywords:** Amyotrophic lateral sclerosis, Molecular neuroscience

## Abstract

Here we report a conserved transcriptomic signature of reduced fatty acid and lipid metabolism gene expression in a *Drosophila* model of *C9orf72* repeat expansion, the most common genetic cause of amyotrophic lateral sclerosis and frontotemporal dementia (ALS/FTD), and in human postmortem ALS spinal cord. We performed lipidomics on C9 ALS/FTD *Drosophila*, induced pluripotent stem (iPS) cell neurons and postmortem FTD brain tissue. This revealed a common and specific reduction in phospholipid species containing polyunsaturated fatty acids (PUFAs). Feeding C9 ALS/FTD flies PUFAs yielded a modest increase in survival. However, increasing PUFA levels specifically in neurons of C9 ALS/FTD flies, by overexpressing fatty acid desaturase enzymes, led to a substantial extension of lifespan. Neuronal overexpression of fatty acid desaturases also suppressed stressor-induced neuronal death in iPS cell neurons of patients with both C9 and TDP-43 ALS/FTD. These data implicate neuronal fatty acid saturation in the pathogenesis of ALS/FTD and suggest that interventions to increase neuronal PUFA levels may be beneficial.

## Abstract (web summary)

Lipidomics revealed that neurons of patients with ALS/FTD have reduced levels of polyunsaturated fatty acid (PUFA)-containing phospholipids. Increasing neuronal PUFA levels increased survival of *Drosophila* models of ALS/FTD and patient neurons, suggesting that interventions that increase neuronal PUFA levels in patients with ALS/FTD may also be beneficial.

## Main

Amyotrophic lateral sclerosis (ALS) and frontotemporal dementia (FTD) are two progressive and invariably fatal neurodegenerative disorders. ALS is characterized by loss of upper and lower motor neurons in the brain and spinal cord, leading to muscle wasting and paralysis, whereas FTD leads to degeneration of the frontal and temporal lobes of the brain, resulting in behavioral and language abnormalities. It is now well established that ALS and FTD represent two ends of a disease continuum, with overlapping clinical and pathological features. ALS and FTD are also linked genetically, with the most common genetic cause of both diseases being an intronic G_4_C_2_ repeat expansion in the *C9orf72* gene (C9 ALS/FTD)^1,2^.

The *C9orf72* repeat is transcribed bidirectionally into sense and antisense repeat RNAs, which are translated into dipeptide repeat proteins (DPRs) by a process termed repeat-associated non-ATG (RAN) translation^3–8^. RAN translation occurs in all reading frames and on both strands to produce five distinct DPR species: poly(GR), poly(GP) and poly(GA) from the sense strand and poly(GP), poly(PR) and poly(PA) from the antisense strand. DPRs and the repetitive RNAs themselves have been implicated in driving neurodegeneration^9–15^. In addition, the repeat expansion leads to reduced levels of the C9orf72 protein^16,17^, which may exacerbate gain-of-function mechanisms^18,19^. Despite numerous cellular pathways implicated downstream of the *C9orf72* repeat expansion since its discovery^20,21^, the molecular mechanisms driving neuronal loss are still unclear.

The brain has the second highest lipid content of any organ in the body, where these molecules serve as critical components of neuronal and organellar membranes. Brain lipids contain a particularly high proportion of polyunsaturated fatty acids (PUFAs)^22^ and epidemiological studies have demonstrated that increased dietary consumption of PUFAs, particularly ω-3 PUFAs, is associated with decreased ALS risk and longer survival after onset^23–25^. However, a molecular understanding of these findings and their relevance to neurodegeneration are unclear. Thus, in the present study, we sought to characterize lipid changes associated with C9 ALS/FTD and understand their contribution to neurodegeneration.

## Results

### Fatty acid and lipid metabolism pathways are decreased in C9 ALS/FTD

**Fig. 1:**
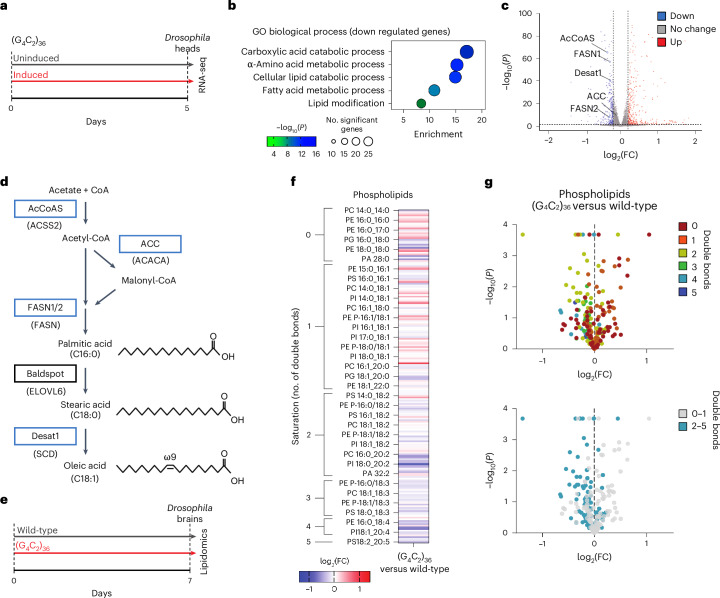
Transcriptomic and lipidomic analyses reveal downregulation of fatty acid and lipid metabolism genes and loss of PUFA-containing phospholipids in C9 flies. **a**, C9 flies were induced for 5 d before performing RNA-seq on heads compared with age-matched uninduced controls. **b**, GO biological process enrichment analyses showing lipid metabolism terms significantly enriched among downregulated genes in RNA-seq comparison of C9-induced fly heads versus uninduced controls (*n* = 4 biological replicates, with 15 fly heads per replicate). Genotype: UAS-(G_4_C_2_)_36_; elavGS. **c**, Volcano plot highlighting significantly downregulated fatty acid synthesis and desaturation genes in C9 fly heads. AcCoAS, Acetyl-coenzyme A synthetase. DEGs in **b** and **c** were calculated with DEseq2 using default parameters (Methods). **d**, Simplified long-chain fatty acid synthesis and desaturation pathway, with *Drosophila* genes in boxes and human orthologous genes in parentheses underneath. The blue boxes indicate genes that were significantly downregulated in C9 fly heads. The ‘C’ number indicates the number of carbons in the fatty acyl chain, the number after the colon denotes the number of double bonds and the ‘ω’ number denotes the position of the final double bond before the methyl carbon. **e**, C9 and wild-type (elavGS driver alone) flies induced for 7 d before brains were dissected for lipidomics analyses. **f**, Heatmap displaying all detected phospholipids as log_2_(fold-change) (log_2_(FC)) over wild-type fly brains (*n* = 3 biological replicates, with 20 fly brains per replicate). **g**, Volcano plots of all detected phospholipid species in C9 fly brains compared with wild-type control flies showing log_2_(fold-change) over wild-type and significance (two-sided Student’s *t*-test). Top, color corresponding to the number of double bonds in the phospholipid species’ most unsaturated fatty acyl chain. Bottom, all PUFA-containing species (two or more double bonds) colored cyan. Genotypes: elavGS, UAS-(G_4_C_2_)_36_; elavGS. Source data

**Extended Data Fig. 1:**
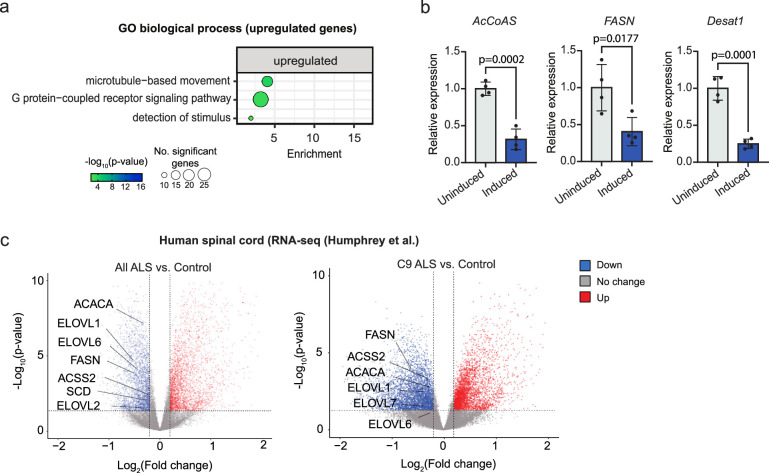
Fatty acid synthesis and desaturation pathway genes are downregulated in C9 ALS post-mortem cervical spinal cord and C9 *Drosophila.* (**a**) GO enrichment of upregulated genes in RNA-seq comparison of C9 fly heads versus controls. Differentially expressed genes were calculated with DEseq2 using default parameters (see Methods). Genotype: UAS-(G_4_C_2_)_36_; elavGS (**b**) Confirmation of C9 *Drosophila* RNA-seq result by RT-qPCR, showing significant downregulation of *AcCoAS, FASN1* and *Desat1* in C9 *Drosophila* heads versus controls, normalised to tubulin (n = 4 biological replicates, with 15 fly heads per replicate). Two-sided, unpaired Students t-test, data presented as mean ± s.d. (**c**) Volcano plots of RNA-seq data from patient post-mortem cervical spinal cord comparing either all ALS (left; n = 138) or just the C9 ALS subset (right; n = 28) with non-neurological disease controls (n = 36) from the New York Genome Center ALS Consortium^26^ highlighting conserved downregulation of canonical fatty acid synthesis and desaturation pathway genes. These data are publicly available at https://rstudio-connect.hpc.mssm.edu/als_spinal_cord_browser/ and all DEGs have also been included in Excel format with this manuscript as Supplementary Data File 1. Source data

To identify pathways dysregulated in neurons in response to expression of the pathological *C9orf72* repeat (C9) expansion, we performed RNA sequencing (RNA-seq) on *Drosophila* heads with 36 G_4_C_2_ repeats expressed exclusively in adult neurons^9^ (Fig. 1a). These experiments were performed at an early timepoint (5 d of repeat expression) to assess early gene expression changes. Gene ontology (GO) enrichment analysis of differentially expressed genes (DEGs) identified only three GO terms enriched among upregulated pathways (Extended Data Fig. 1a). However, among the most significantly downregulated pathways, we observed multiple terms related to fatty acid and lipid metabolism (Fig. 1b). This included reduction of several genes throughout the canonical long-chain fatty acid synthesis and desaturation pathway, such as *AcCoAS*, *FASN1*, *FASN2* and *Desat1* (Fig. 1c,d and Extended Data Fig. 1b). To determine whether these lipid gene expression changes were conserved in human disease, we reanalyzed the largest bulk RNA-seq dataset generated from ALS postmortem spinal cords, comprising 138 cases of ALS and 36 non-neurological disease controls^26^. Strikingly, genes in the same lipid and fatty acid metabolism pathway were also downregulated in ALS spinal cords, including *ACACA*, *ACSS2*, *FASN*, *ELOVL6* and *SCD* (orthologous to *Drosophila ACC*, *AcCoAS*, *FASN1*/*FASN2*, *Baldspot* and *Desat1*, respectively) (Extended Data Fig. 1c). These genes were similarly downregulated in the subset of 28 spinal cords of patients with C9 ALS present within the dataset (Extended Data Fig. 1c). Together, these findings demonstrate conserved transcriptional dysregulation of lipid metabolism, and specifically downregulation of fatty acid synthesis and desaturation processes, in ALS/FTD neurons.

### Altered phospholipid saturation in *C9orf72* flies and iPS cell neurons

**Extended Data Fig. 2:**
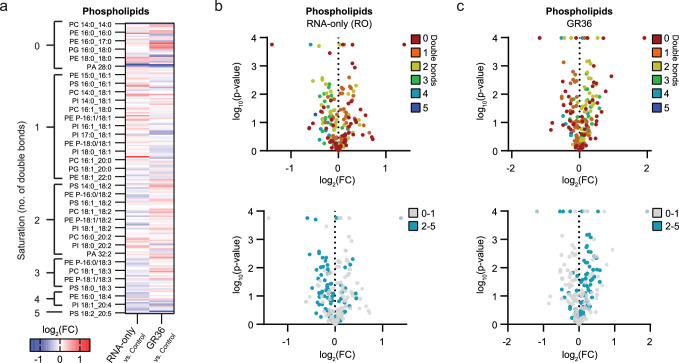
Lipidomic analyses in RNA-only (RO) and GR36 fly brains. (**a**) Heatmap displaying all detected phospholipids as log_2_(fold-change) over control fly brains (n = 3 biological replicates per condition with 15 fly brains per replicate). Lipids were normalized by lipid class. (**b, c**) Volcano plots of all detected phospholipid species in RO (**b**) or GR36 (**c**) fly brains compared to wild-type control flies. Values represent log_2_(fold-change) over control and significance (two-sided Student’s t-test) across all replicates within the labeled group. In top plot, color corresponds to the number of double bonds in the phospholipid species’ most unsaturated fatty acyl chain. In bottom, PUFA-containing species (≥2 double bonds) are highlighted in blue. Genotypes: elavGS, UAS-(G_4_C_2_)_36_ RO; elavGS, UAS-(GR)_36_; elavGS. Source data

In light of the dysregulation of the fatty acid and lipid metabolism transcriptional signature, we next determined whether lipids were altered in C9 fly brains. We dissected brains from C9 and wild-type control flies after 7 d of repeat expression and conducted lipidomic analysis of >1,400 complex lipid species (Fig. 1e). Among the different lipid classes that were measured, these experiments revealed a consistent change across phospholipid species (Fig. 1f,g). Phospholipids are composed of two fatty acyl chains and a head group. The number of double bonds in each chain determines their saturation with zero double bonds being a completely saturated fatty acid (SFA) and each additional double bond increasing unsaturation. Species with two or more double bonds are classed as PUFAs. We observed a marked shift toward higher phospholipid saturation and loss of PUFA-containing phospholipids compared with control brains (Fig. 1f,g). To test whether this phenotype was being driven by repeat RNA or DPRs, we then conducted lipidomics on brains from RNA-only (RO) flies, where the repeat is interrupted by stop codons and does not produce DPRs, and GR36 flies, in which the repeat sequence is codon optimized to no longer produce G_4_C_2_ RNAs but does produce toxic poly(GR) through ATG-driven translation^9,27^. We found that RO flies, but not GR36 flies, recapitulate the reduction in PUFA-containing phospholipids observed in C9 fly brains (Extended Data Fig. 2a–c). This indicates that reduction in phospholipid unsaturation is driven by repeat RNA rather than DPRs.

**Fig. 2:**
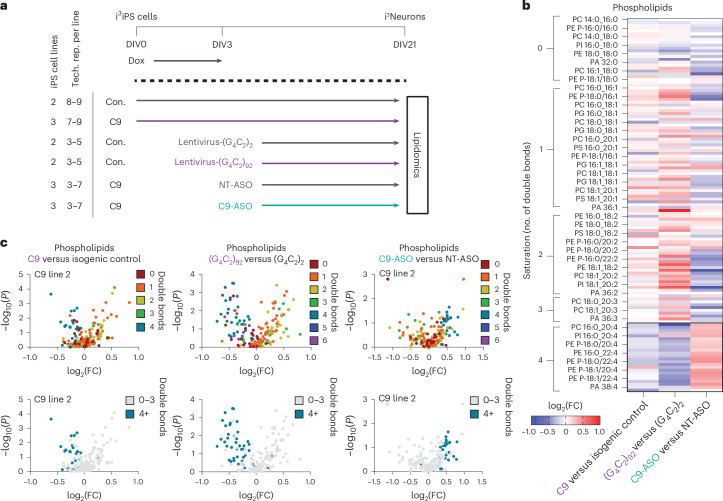
C9 repeats cause loss of highly unsaturated phospholipid species in iPS-cell-derived neurons. **a**, C9 and isogenic control i^3^iPS cells were induced using the i^3^Neuron protocol^28,29^ and cultured for 21 d in vitro (DIV21) for lipidomic analyses. To confirm disease specificity of lipid changes, control (Con.) lines were transduced with (G_4_C_2_)_92_ repeat or (G_4_C_2_)_2_ control lentiviruses, and C9 lines were treated with a *C9orf72* ASO or an NT control ASO^7^. Tech. rep., Technical replicate. **b**, Heatmap displaying all detected phospholipids as log_2_(fold-change) over controls for each experimental condition (*n* = 3 C9 lines, *n* = 2 control lines + lentiviruses, *n* = 3 C9 lines + ASO). Highly unsaturated species (four or more double bonds, outlined) were reduced in C9 lines and after lentivirus-(G_4_C_2_)_92_ treatment but increased in C9 lines treated with the C9 ASO compared with C9 lines treated with the NT ASO, indicating that these changes were driven by C9 repeats. The gray boxes indicate phospholipid species that were outside the fold-change range. **c**, Volcano plots of all detected phospholipid species in each line or condition compared with its control, displaying downregulation of highly unsaturated species (four or more double bonds). Values represent log_2_(fold-change) over control and significance (two-sided Student’s *t*-test) across all replicates within the labeled group. Top, color corresponding to the number of double bonds in the phospholipid species’ most unsaturated fatty acyl chain. Bottom, the highly unsaturated species (four or more double bonds) highlighted in blue. Source data

**Extended Data Fig. 3:**
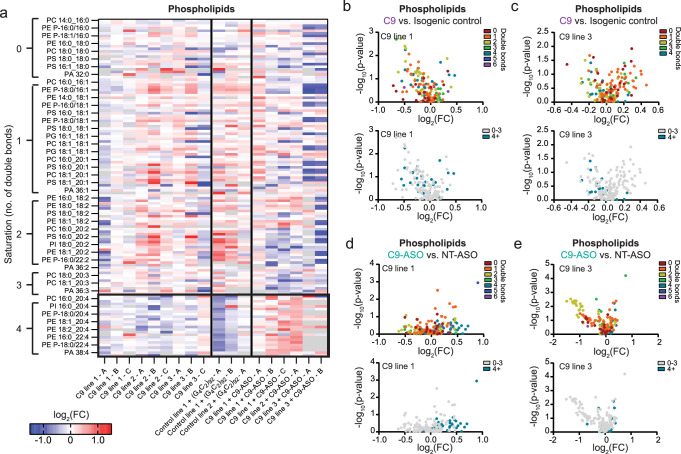
Phospholipid levels in i^3^Neurons, displayed as separate neuronal inductions/lines. (**a**) Heatmap displaying all detected phospholipids as log_2_(fold-change) over control in each neuronal induction separately. Lipids are normalized by lipid class. Grey boxes indicate phospholipid species that are outside the fold-change range or were not detected in that induction. Loss of highly unsaturated species is consistently observed across neuronal inductions in C9 lines and control lines expressing 92-repeats, while phenotype is prevented by C9-ASO treatment. (**b, c**) Volcano plots of all detected phospholipid species in C9 lines 1 (**b**) and 3 (**c**) compared to their individual isogenic control lines, displaying downregulation of highly unsaturated species (≥4 double bonds). (**d, e**) Volcano plots displaying all phospholipid species in C9 lines 1 (**d**) and 3 (**e**) treated with a C9-ASO compared to a NT-ASO control. In (b-e), values represent log_2_(fold-change) over control and significance (two-sided Student’s t-test) across all replicates/inductions within the labeled group. In top plots, color corresponds to the number of double bonds in the phospholipid species’ most unsaturated fatty acyl chain. Source data

**Extended Data Fig. 4:**
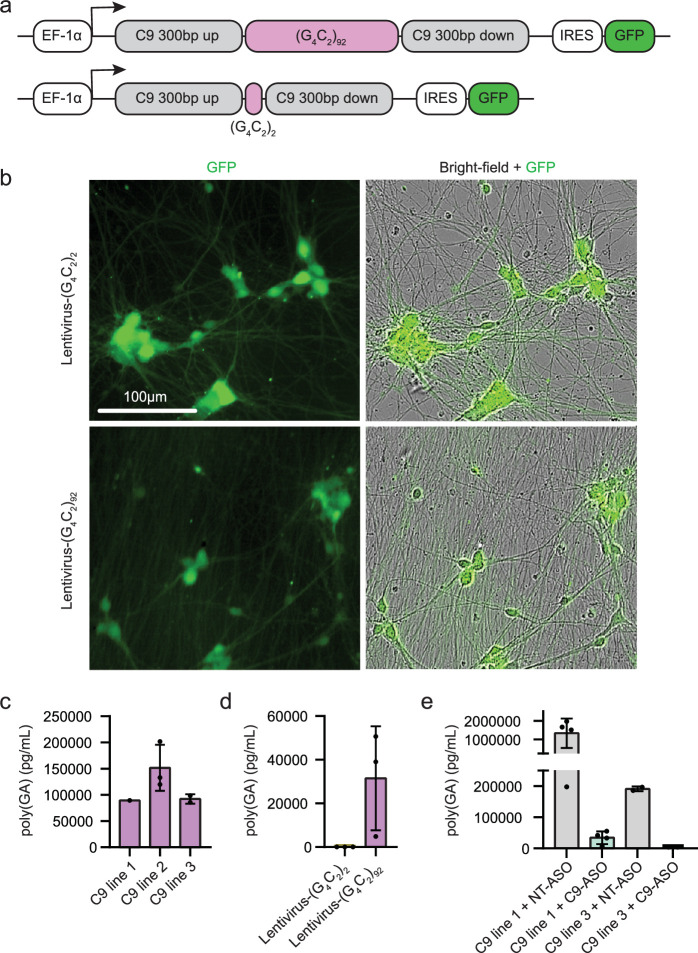
Poly(GA) levels in i^3^Neuron lines treated with (G_4_C_2_) lentiviruses or sense repeat-targeted antisense oligonucleotides (ASOs). (**a**) Lentiviral 92 (Lentivirus-(G_4_C_2_)_92_) and 2 (Lentivirus-(G_4_C2)_2_) repeat constructs have 300 bp of endogenous repeat-flanking sequence to facilitate RAN translation and include an IRES-GFP for live-cell visualization. (**b**) (G_4_C_2_)_92_ and (G_4_C_2_)_2_ lentiviruses were titrated via GFP signal (live imaged) to high transduction efficiencies for lipidomic experiments. (c-e) poly(GA) immunoassay in (**c**) C9 lines (n = 1–3 inductions per line), (**d**) control lines treated with (G_4_C_2_)_92_ or (G_4_C_2_)_2_ (n = 3 inductions per line), and (**e**) C9 lines treated with sense repeat-targeted (C9) ASOs or non-targeted (NT) control ASOs from^7^ (n = 2–4 inductions per line). All measurements were taken on DIV21 from the same neuronal inductions used for lipidomic analyses. Each dot is an independent induction. Error bars show ± s.d. Source data

**Extended Data Fig. 5:**
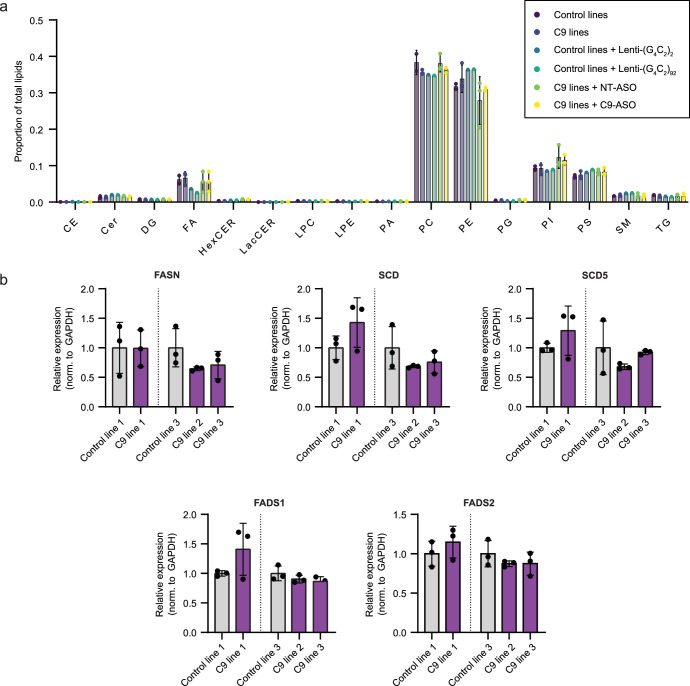
Lipid class distributions and lipid gene qPCRs in i^3^Neurons. (**a**) Lipid classes displayed as proportion of total lipidome in i^3^Neurons. Data points represent average lipid class level for each separate i^3^Neuron line (biological replicate), averaged across inductions (n = 2 control lines; n = 3 C9 lines; n = 3 C9 lines + NT-ASO; n = 3 C9 lines + C9-ASO). For lentiviral experiments, biological replicates were considered separate inductions, and the single datapoints in this figure represent the average of n = 3 inductions per virus. CE = cholesterol ester; Cer = ceramide; DG = diacylglyceride; FA = fatty acid; HexCER = hexosylceramide; LacCER = lactosylceramides; LPC = lysophosphatidylcholine; LPE = lysophosphatidylethanolamine; PA = phosphatidic acid; PC = phosphatidylcholine; PE = phosphatidylethanolamine; PG = phosphatidylglycerol; PI = phosphatidylinositol; PS; phosphatidylserine; SM = sphingomyelin; TG = triacylglyceride. (**b**) RT-qPCRs of fatty acid synthase (*FASN*) and neuronal desaturases, showing no significant changes in C9 compared to isogenic controls (One-way ANOVA for control line 3 and C9 lines 2 and 3; two-sided unpaired Student’s t-test for control line 1 and C9 line 1; no significant differences found). Error bars show mean ± s.d. Source data

To determine whether these lipidomic alterations are conserved in a human model, we performed lipidomic analyses on C9 repeat-containing iPS cell cortical neurons and isogenic controls, which were induced with the i^3^Neuron protocol^28,29^ and collected 21 d later (Fig. 2a). As in the C9 flies, we observed a striking shift toward higher phospholipid saturation and loss of highly polyunsaturated phospholipids (containing fatty acyl chains with four or more double bonds) compared with controls (Fig. 2b,c and Extended Data Fig. 3a–c). To confirm that these changes were driven by the C9 repeat expansion and not cell-line variability, or other mechanisms such as *C9orf72* loss of function, we next performed lipidomic analyses in two cross-validation experiments (Fig. 2a). In the first, we exogenously expressed C9 repeats in control iPS cell neurons by transducing with (G_4_C_2_)_92_ or (G_4_C_2_)_2_ lentiviruses. As expected, lentiviral repeat expression resulted in substantial DPR production in (G_4_C_2_)_92_- but not (G_4_C_2_)_2_-transduced neurons (Extended Data Fig. 4). Exogenous repeat expression in control iPS cell neurons recapitulated the loss of highly polyunsaturated phospholipids that we observed in the C9 patient lines (Fig. 2b,c). This shows that expression of expanded *C9orf72* repeats is sufficient to drive the lipid changes observed. Next, we treated our three C9 lines with an antisense oligonucleotide (ASO) that specifically targets transcripts containing the C9 repeat^7^. This led to an almost complete (>95%) reduction in DPRs compared with a nontargeting (NT) control, confirming effective knockdown (Extended Data Fig. 4). C9 repeat knockdown prevented the reduction in highly polyunsaturated phospholipids that we observed in the C9 patient lines, suggesting that the C9 repeat was driving these changes (Fig. 2b,c and Extended Data Fig. 3a,d,e). Lipid class proportionality was similar across conditions, suggesting that these observations are the result of a specific shift in phospholipid saturation rather than a global alteration in lipid class abundance (Extended Data Fig. 5a). Together, these results demonstrate a striking and specific decrease in PUFA-containing phospholipid species caused by the presence of expanded C9 repeats.

Given our observation of conserved phospholipid saturation alterations in i^3^Neurons, we wondered whether this was being driven by an alteration in neuronal desaturase expression as observed in our C9 flies. We assayed RNA levels of *FASN*, as well as the four major neuronal lipid desaturases, *SCD*, *SCD5*, *FADS1* and *FADS2*, in DIV21 i^3^Neurons, but found no significant changes in any of these genes compared with isogenic control lines at this timepoint (Extended Data Fig. 5b). This suggests that either desaturase expression is altered at an earlier timepoint than we assayed here or alternative upstream mechanisms are driving the observed lipidomic saturation shifts in C9 i^3^Neurons.

### Altered phospholipid saturation in FTLD postmortem frontal cortex

**Fig. 3:**
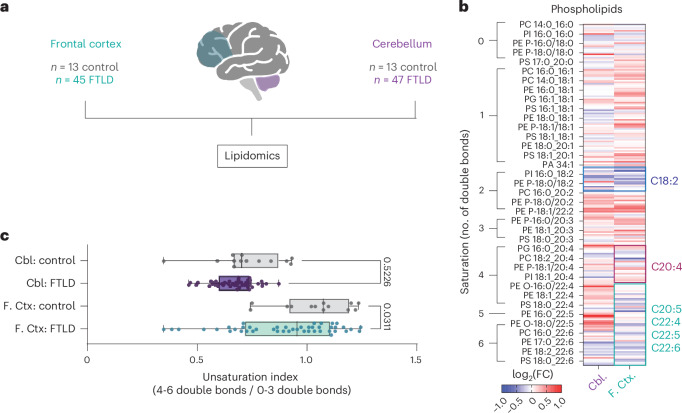
Highly unsaturated phospholipids are decreased in FTLD postmortem frontal cortex. **a**, Lipidomics performed on postmortem tissues from FTLD and age- and sex-matched control frontal cortex and cerebellum samples. **b**, Heatmap displaying all detected phospholipids as log_2_(fold-change) over control in each brain region. Highly unsaturated species (four or more double bonds, except those containing C20:4) are outlined in cyan and show broad downregulation in FTLD frontal cortex but not cerebellum. Species containing arachidonic acid (C20:4) are outlined in red and many display upregulation in FTLD frontal cortex. Species containing linoleic acid (C18:2) are outlined in blue and show downregulation in both tissue regions. Cbl, cerebellum; F. Ctx, frontal cortex. **c**, Unsaturation indices of phospholipids from FTLD and control brain regions, demonstrating a significant reduction in frontal cortex but not in cerebellum (two-way ANOVA, main effects of brain region (*P* < 0.0001) and disease state (*P* = 0.0154); post-hoc comparisons of FTLD versus control *P* values adjusted with Šídák’s multiple-comparison test; *n* = 13 control and *n* = 45 FTLD frontal cortex; *n* = 13 control and *n* = 47 FTLD cerebellum samples from separate individuals). The bounds of the box represent the 25th and 75th percentiles, whereas the whiskers represent minima and maxima and the line the median. Schematic in **a** created using BioRender; Cammack, A. (2024) https://BioRender.com/b41l552. Source data

**Extended Data Fig. 6:**
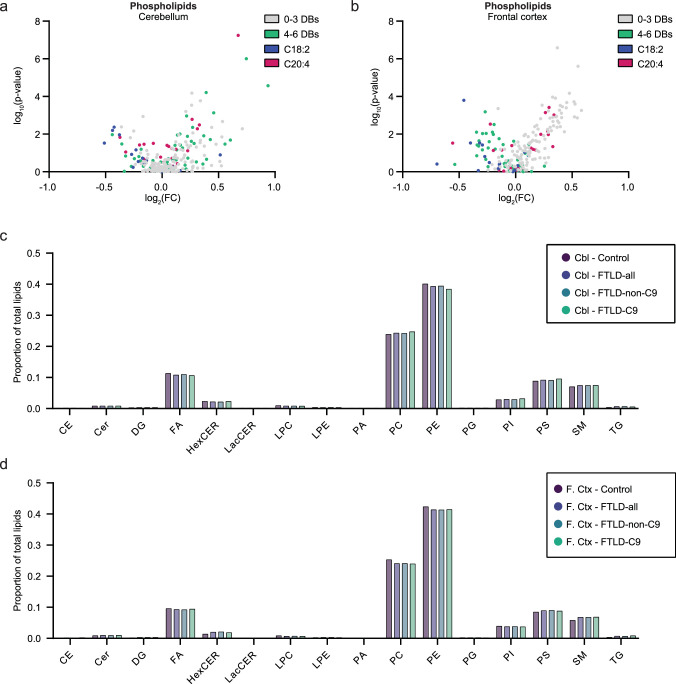
Volcano plots of phospholipid species and lipid class distributions in FTLD versus control post-mortem tissues. (**a, b**) Volcano plots of all detected phospholipid species in FTLD compared to non-neurological control in cerebellum (**a**) and frontal cortex (**b**), displaying downregulation of highly unsaturated species (≥4 double bonds) in the frontal cortex. Values represent log_2_(fold-change) over control and significance (two-sided Student’s t-test). (**c, d**) Lipid classes displayed as proportion of total lipidome in control and FTLD post-mortem cerebellum (**c**) and frontal cortex (**d**). Bars represent average across all samples (n = 45–47 FTLD, n = 13 control). CE = cholesterol ester; Cer = ceramide; DG = diacylglyceride; FA = fatty acid; HexCER = hexosylceramide; LacCER = lactosylceramides; LPC = lysophosphatidylcholine; LPE = lysophosphatidylethanolamine; PA = phosphatidic acid; PC = phosphatidylcholine; PE = phosphatidylethanolamine; PG = phosphatidylglycerol; PI = phosphatidylinositol; PS; phosphatidylserine; SM = sphingomyelin; TG = triacylglyceride. Source data

We next asked whether phospholipid saturation dysregulation is also present in human disease tissue. We performed lipidomic analyses on postmortem affected (frontal cortex) and less affected (cerebellum) brain tissue from a large cohort of 47 individuals with neuropathologically confirmed FTD, termed frontotemporal lobar degeneration (FTLD), 15 of whom had a C9 mutation and 13 age- and sex-matched, healthy controls (Fig. 3a). In concordance with our fly and iPS cell-neuron data, in FTLD frontal cortex we observed a decrease in highly unsaturated phospholipids, particularly those containing four or more double bonds in their most unsaturated fatty acyl chain (Fig. 3b,c and Extended Data Fig. 6a,b), whereas lipid class proportionality was similar between control and FTLD tissue in both brain regions (Extended Data Fig. 6c,d). It is interesting that there was one exception, in species containing arachidonic acid (C20:4), some of which were upregulated in FTLD tissues. This is consistent with the association of arachidonic acid with inflammatory signaling^30^, highlighting additional lipid alterations that occur in end-stage disease, as well as a previous study showing elevated arachidonic acid in C9 repeat disease models^31^. These changes in highly polyunsaturated phospholipids were largely specific to the frontal cortex. In both tissue regions, we also observed a decrease in species containing linoleic acid (C18:2), an essential PUFA that is a precursor for highly unsaturated fatty acid species^32^. Thus, consistent with C9 flies and iPS cell neurons, FTLD postmortem brains displayed a reduction in highly polyunsaturated species, specifically in the affected region.

### Promoting neuronal fatty acid desaturation increases C9 fly survival

**Fig. 4:**
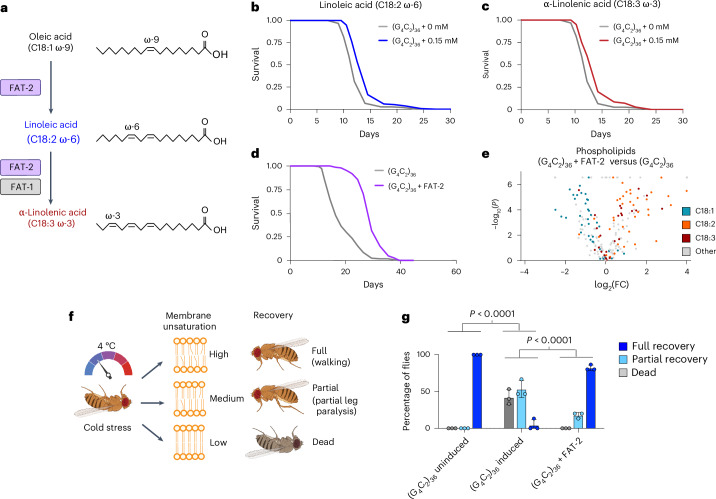
Promoting fatty acid desaturation through either genetic or feeding paradigms extends C9 fly survival and prevents cold-stress-induced death and paralysis. **a**, Simplified fatty acid desaturation pathway. **b**,**c**, Dietary supplementation of linoleic (**b**) or α-linolenic (**c**) acid at 0.15 mM extended C9 fly survival (linoleic acid *P* = 5.687 × 10^−5^; α-linolenic acid *P* = 2.951 × 10^−6^, log-rank test; *n* = 152 (0 mM), *n* = 134 (0.15 mM C18:2), *n* = 141 (0.15 mM C18:3)). **d**, Neuronal overexpression of *FAT-2* extended C9 fly survival (*P* = 5.119 × 10^−38^), log-rank test, *n* = 147 ((G_4_C_2_)_36_), *n* = 146 ((G_4_C_2_)_36_) + FAT-2. **e**, Volcano plot showing neuronal expression of *FAT-2* resulting in conversion of C18:1 into C18:2 and C18:3 within C9 fly brain phospholipids (*n* = 3 biological replicates, with 20 fly brains per replicate). Values represent log_2_(fold-change) over control and significance (two-sided Student’s *t*-test) across all replicates within the labeled group. **f**, Schematic diagram of cold stress assay. **g**, C9 flies show sensitivity to cold stress, with significantly increased death and paralysis, and decreased recovery compared with uninduced controls (*P* < 0.0001). Neuron-specific overexpression of *Desat1* or *FAT-2* in C9 flies significantly increased the proportion of flies experiencing a full recovery compared with (G_4_C_2_)_36_ alone and significantly reduced death post-exposure compared with (G_4_C_2_)_36_ alone (*n* = 3 biological replicates, containing 15 flies per replicate). Results were analyzed by *χ*^2^ test. Data are presented as mean ± s.d. Genotypes: UAS-(G_4_C_2_)_36_; elavGS, UAS-(G_4_C_2_)_36_; elavGS/UAS-Desat1, UAS-(G_4_C_2_)_36_; elavGS/UAS-FAT-2. Schematic in **f** created using BioRender; Isaacs, A. (2025) https://BioRender.com/v04i884. Source data

**Extended Data Fig. 7:**
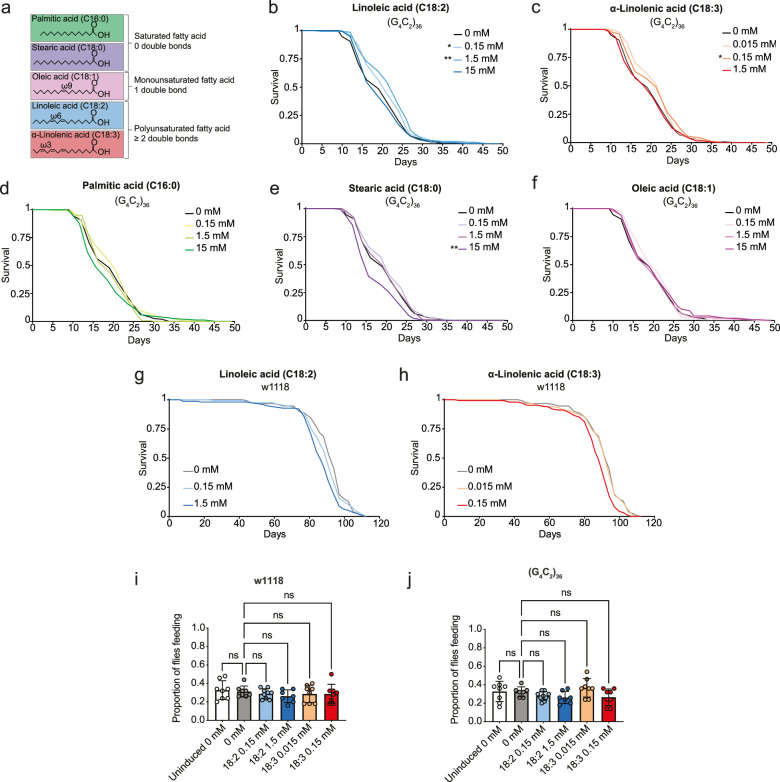
Lifespans of C9 flies fed saturated, monounsaturated, and polyunsaturated long chain fatty acids. (**a**) Structure and saturation of selected fatty acids. (**b**) Linoleic acid significantly increased survival of C9 flies at 0.15 mM (*P* = 0.031) and 1.5 mM (*P* = 0.001) concentrations, but not at 15 mM (*P* = 0.699). n = 148 (0 mM), n = 151 (C18:2 0.15 mM), n = 144 (C18:2 1.5 mM), n = 135 (C18:2 15 mM). Log-rank test used for all comparisons. (**c**) α-linolenic acid significantly increased survival of C9 flies at 0.015 mM (*P* = 0.048) and 0.15 mM (*P* = 0.044) concentrations, but not at 1.5 mM (*P* = 0.495). Log-rank test used for all comparisons. (**d**) Palmitic acid had no significant effect on survival of C9 flies at any of the concentrations tested (0.15 mM, *P* = 0.052, 1.5 mM *P* = 0.182, 15 mM *P* = 0.473). n = 148 (0 mM), n = 145 (C16:0 0.15 mM), n = 146 (C16:0 1.5 mM), n = 147 (C16:0 15 mM). Log-rank test used for all comparisons. (**e**) Stearic acid had no significant effect at 0.15 mM (*P* = 0.079) or 1.5 mM (*P* = 0.992), but significantly decreased survival at 15 mM (*P* = 0.008). n = 148 (0 mM), n = 146 (C18:0 0.15 mM), n = 149 (C18:0 1.5 mM), n = 150 (C18:0 15 mM). Log-rank test used for all comparisons. (**f**) Oleic acid had no significant effect on survival at any of the concentrations tested (0.15 mM, *P* = 0.285, 1.5 mM *P* = 0.782, 15 mM *P* = 0.186). n = 148 (0 mM), n = 143 (C18:1 0.15 mM), n = 142 (C18:1 1.5 mM), n = 150 (C18:1 15 mM). Log-rank test used for all comparisons. Genotype: UAS-(G_4_C_2_)_36_; elavGS. (**g**) Linoleic acid supplementation had no effect on wild-type lifespan at 0.15 mM (*P* = 0.162) and significantly shortened wild-type lifespan at the 1.5 mM (*P* = 4.352×10^−5^) concentration. n = 150 (0 mM), n = 151 (C18:2 0.15 mM), n = 152 (C18:2 1.5 mM). Log-rank test used for all comparisons. (**h**) α-linolenic acid had no effect on wild-type lifespan at 0.015 mM (*P* = 0.599), and significantly shortened wild-type lifespan at the 0.05 mM concentration (*P* = 6.22×10^−6^). n = 150 (0 mM), n = 150 (C18:3 0.015 mM), n = 148 (C18:3 0.15 mM). Log-rank test used for all comparisons. (**i, j**) Food supplementation with linoleic or α-linolenic acid does not alter proboscis extension response of wild-type (**i**) or C9 (**j**) flies. Flies were placed onto new food 24 hours before assay was performed, at a density of five flies per biological replicate vial. All groups were induced with RU486 except for the uninduced conditions. Two-way ANOVA with Tukey’s multiple comparison test was used to calculate statistical significance. Data presented as mean ± s.d. (**i**) n = 7 flies (18:2 1.5 mM) n = 8 flies (uninduced 0 mM, 0 mM, 18:3 0.015 mM, 18:3 0.15 mM), n = 9 flies (18:2 0.15 mM). (**j**) n = 7 flies (0 mM, 18:2 1.5 mM) n = 8 flies (uninduced 0 mM, 18:3 0.015 mM, 18:3 0.15 mM), n = 9 flies (18:2 0.15 mM). Genotypes: elavGS, UAS-(G_4_C_2_)_36_; elavGS. Source data

To determine whether dysregulated lipid metabolism directly contributes to neurotoxicity, we first asked whether dietary supplementation with fatty acids could rescue survival of C9 flies. The PUFAs linoleic acid (C18:2) and α-linolenic acid (C18:3) (Fig. 4a) significantly but modestly extended median survival of C9 flies by 12–15% (Fig. 4b,c and Extended Data Fig. 7a–c), whereas adding saturated or monounsaturated fatty acid species (palmitic acid (C16:0), stearic acid (C18:0) and oleic acid (C18:1)) had either no effect or decreased survival (Extended Data Fig. 7d–f). The extensions in survival generated by PUFA supplementation were specific to the disease model, because supplementing wild-type flies with linoleic acid or α-linolenic acid either decreased or had no effect on wild-type survival (Extended Data Fig. 7g,h). Furthermore, these rescues were not the result of an alteration in feeding behavior, as measured by the proboscis extension assay (Extended Data Fig. 7i,j).

**Extended Data Fig. 8:**
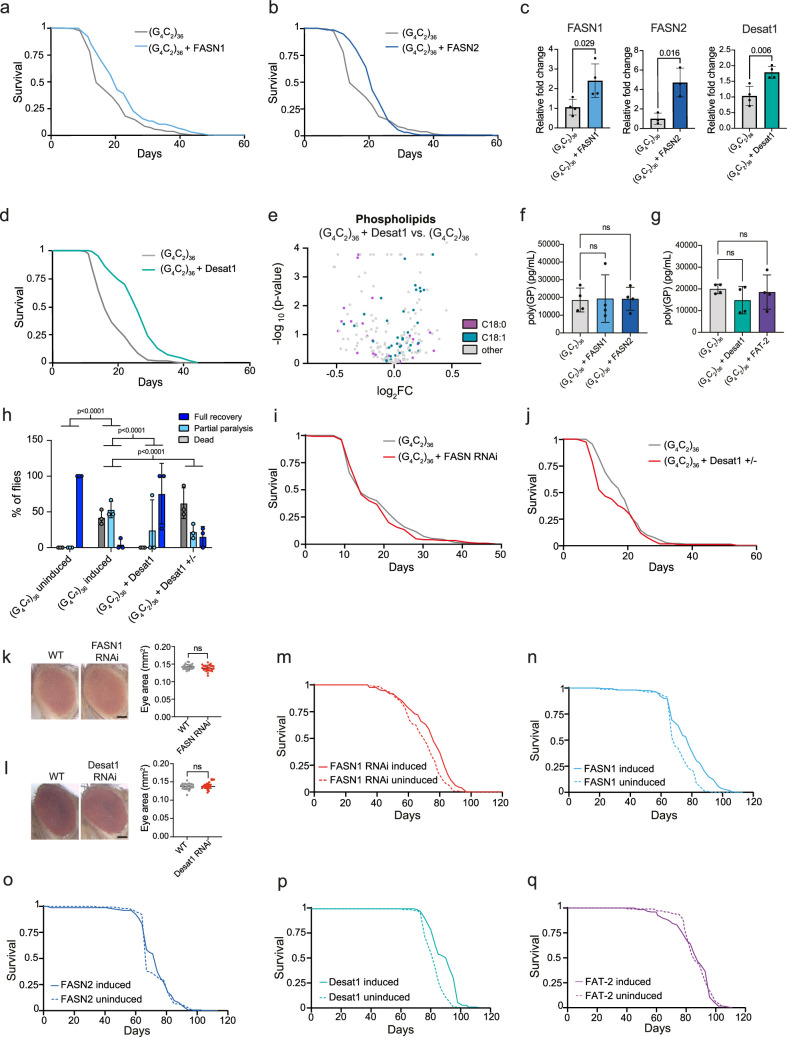
Overexpression of fatty acid synthases extends C9 survival, while fatty acid synthase and desaturase overexpression does not alter poly(GP) levels. (**a, b**) Neuronal overexpression of *FASN1* (**a**) or *FASN2* (**b**) extended C9 fly survival (*FASN1* *P* = 1.16×10^−5^; *FASN2* *P* = 0.003), log-rank test used for each comparison. n = 151 ((G_4_C_2_)_36_), n = 144 ((G_4_C_2_)_36_ + FASN1), n = 132 (G_4_C_2_)_36_ + FASN2. (**c**) Confirmation of *FASN1*, *FASN2* and *Desat1* overexpression in (G_4_C_2_)_36_ fly heads after 7 days of neuronal expression (n = 4 biological replicates with 15 heads per replicate). Two-sided, unpaired Students t-test, data presented as mean ± s.d. (**d**) Neuronal overexpression of *Desat1* extended C9 fly survival (*P* = 5.839×10^−20^). n = 147 ((G_4_C_2_)_36_), n = 143 ((G_4_C_2_)_36_ + Desat1), log-rank test. (**e**) Neuronal expression of *Desat1* results in conversion of C18:0 into C18:1 in phospholipids from dissected C9 fly brains. Lipids normalized by lipid class. Values represent log_2_(fold-change) over control and significance (two-sided Student’s t-test). (**f**) Neuronal expression of *FASN1* (*P* = 0.989) or *FASN2* (*P* = 0.992) did not alter poly(GP) levels in C9 fly heads. One-way ANOVA, followed by Tukey’s post-hoc test. n = 4 biological replicates per condition, consisting of 10 heads per replicate. Data presented as mean ± s.d. (**g**) Neuronal expression of *Desat1* (*P* = 0.401) or *FAT-2* (*P* = 0.920) did not alter poly(GP) levels in C9 fly heads. One-way ANOVA, followed by Tukey’s post-hoc test. n = 4 biological replicates per condition, consisting of 10 heads per replicate. Data presented as mean ± s.d. (**h**) *Desat1* neuronal overexpression significantly increased the proportion of flies experiencing a full recovery compared to (G_4_C_2_)_36_ alone, whereas loss of one copy of *Desat1* in C9 flies significantly increased death and partial paralysis after cold stress. n = 3 biological replicates, containing 15 flies per replicate vial. Results were analyzed by Chi-square test. Data presented as mean ± s.d. Note that the (G_4_C_2_)_36_; elavGS uninduced and induced data are the same as in Fig. 4g. Genotypes (a-h): UAS-(G_4_C_2_)_36_; elavGS, UAS-(G_4_C_2_)_36_; elavGS/UAS-Desat1, UAS-(G_4_C_2_)_36_; elavGS/UAS-Desat1[42]. (**i**) Knocking down *FASN1* in neurons of (G_4_C_2_)_36_ flies does not significantly alter C9 fly survival (*P* = 0.767), n = 124 ((G_4_C_2_)_36_), n = 136 ((G_4_C_2_)_36+_ FASN RNAi), log-rank test. (**j**) Loss of one copy of *Desat1* significantly worsens C9 fly survival (*P* = 0.016), n = 154 ((G_4_C_2_)_36_), n = 148 ((G_4_C_2_)_36_ + Desat1 +/−), log-rank test. (**k, l**) Knocking down *FASN1* (*P* = 0.127) (**k**) or *Desat1* (*P* = 0.984) (**l**) in wild-type eye neurons does not cause neurodegeneration. Two-sided, unpaired Students t-test, n = 25 biological replicates per genotype, data presented as mean ± s.d. Scale bars represent 0.1 mm. Genotypes (**k, l**): GMR-Gal4, GMR-Gal4; UAS-FASN RNAi, GMR-Gal4; UAS-Desat1 RNAi. Genotypes: GMR-Gal4, GMR-Gal4; UAS-FASN RNAi, GMR-Gal4; UAS-Desat1 RNAi. (**m**) Knocking down *FASN1* in wild-type neurons slightly increases lifespan (*P* = 0.003), log-rank test, n = 133 (FASN1 RNAi induced), n = 148 FASN1 RNAi uninduced. Genotype: UAS-FASN1 RNAi; elavGS. (**n**) *FASN1* overexpression in neurons of wild-type flies extended lifespan (*P* = 3.386×10^−8^), log-rank test, n = 156 (FASN1 induced), n = 151 (FASN1 uninduced). (**o**) *FASN2* overexpression in neurons of wild-type flies had no effect on lifespan (*P* = 0.866), log-rank test, n = 156 (FASN2 induced), n = 141 (FASN2 uninduced). (**p**) *Desat1* overexpression in neurons of wild-type flies extended lifespan (*P* = 1.567×10^−10^), log-rank test, n = 114 (Desat1 induced), n = 116 (Desat1 uninduced). (**q**) Overexpression of *FAT-2* in neurons of wild-type flies had no effect on lifespan (*P* = 0.590), log-rank test, n = 123 (FAT-2 induced), n = 105 (FAT-2 uninduced). Genotypes (n-q): elavGS, UAS-FASN1; elavGS, UAS-FASN2; elavGS, elavGS/UAS-Desat1, elavGS/UAS-FAT-2. Source data

As the rescue with feeding PUFAs was modest, we next asked whether neuronal overexpression of fatty acid synthase or desaturase genes, which encode enzymes that produce and desaturate long-chain fatty acids, respectively (Fig. 4a), could prevent C9-associated neurodegeneration in vivo. We crossed our C9 flies to flies overexpressing lipid pathway genes, using the same adult neuronal driver as the C9 repeats, and measured survival. Although overexpression of the fatty acid synthase genes *FASN1* and *FASN2* resulted in survival extensions (Extended Data Fig. 8a–c), the most impressive rescues occurred when overexpressing fatty acid desaturases. Overexpression of *Desat1*, which introduces a double bond into the acyl chain of saturated fatty acids (for example, C18:0) to produce the monounsaturated fatty acid oleic acid (C18:1) significantly extended C9 fly survival, increasing median survival from 15 d to 25 d, an increase of 67% (Extended Data Fig. 8d). Linoleic acid (C18:2) and α-linolenic acid (C18:3) are termed essential fatty acids, because these species cannot be synthesized endogenously by most animals, including *Drosophila* and humans, and must be obtained from the diet to serve as precursors for generating more highly unsaturated PUFAs. However, the nematode *Caenorhabditis elegans* does possess fatty acid desaturases capable of endogenously synthesizing these essential PUFAs from more saturated precursors. Neuronal expression in C9 *Drosophila* of *C. elegans FAT-2*, a Δ12/Δ15 fatty acid desaturase that produces linoleic acid and α-linolenic acid from monounsaturated fatty acids^33,34^, provided an even greater rescue, extending median survival from 15 d to 27.5 d, an increase of 83% (Fig. 4d). We confirmed that both desaturases modified lipid saturation in the expected way, with *Desat1* converting C18:0 to C18:1 (Extended Data Fig. 8e) and *FAT-2* causing a marked conversion of C18:1 to C18:2 and C18:3 in C9 fly brains (Fig. 4e). Importantly, these genetic rescues were not the result of an effect on DPR levels, because poly(GP) levels in C9 fly heads were unchanged by overexpression of any lipid-related genes (Extended Data Fig. 8f,g).

Lipid saturation influences the biophysical properties of cellular membranes, particularly the packing of membrane phospholipids, with increased unsaturation resulting in an increase in membrane fluidity^33,35,36^. As membrane fluidity increases with temperature, poikilothermic *Drosophila* must adjust their membrane lipid content on temperature fluctuations to survive^37,38^. Indeed, *Drosophila* alter their feeding preferences in response to cold exposure to incorporate more PUFAs into their lipid bilayers to maintain their membrane fluidity^38^. Therefore, to investigate the mechanism by which neuronal desaturase expression is beneficial and whether membrane fluidity plays a role, we used a *Drosophila* cold-stress membrane fluidity paradigm. C9 flies were exposed to 4 °C for 18 h, which causes a cold-induced paralysis attributable to decreased membrane fluidity^33,39,40^, and then returned to room temperature, with recovery scored 1 h later (Fig. 4f). Whereas all nonrepeat-expressing control flies showed a full recovery after this period, 42% of flies expressing (G_4_C_2_)_36_ were dead and 54% were partially paralyzed, with only 2% exhibiting a full recovery (Fig. 4g). Strikingly, overexpressing either *Desat1* or *FAT-2* specifically in neurons prevented death entirely after cold exposure in C9 flies and resulted in a dramatically improved recovery (Fig. 4g). These data suggest that an increase in membrane fluidity contributes to the beneficial effect of neuronal desaturase overexpression.

We next asked whether loss of fatty acid synthesis or desaturation exacerbates neurodegeneration or is sufficient to induce neurodegeneration on its own. Knocking down *FASN1* in neurons did not alter C9 fly survival, however, expressing a *Desat1* hypomorphic mutant allele exacerbated toxicity in C9 flies, reducing survival and worsening tolerance in the cold-stress assay (Extended Data Fig. 8h–j). We then tested whether loss or overexpression of these genes can modify lifespan and neurodegeneration in wild-type flies. Knocking down *FASN1* or *Desat1* in eye neurons of wild-type flies using a GMR-GAL4 driver was not sufficient to induce neurodegeneration (Extended Data Fig. 8k,l) and *FASN1* knockdown in all neurons did not significantly modify wild-type fly lifespan (Extended Data Fig. 8m), suggesting that loss of these genes is insufficient to induce neurodegeneration on its own. Similarly, overexpressing these genes in neurons of healthy control flies had either no effect on survival or increased survival by a much smaller magnitude than that observed in the context of C9 repeats (Extended Data Fig. 8n–q). Thus, neuronal fatty acid desaturation alterations appear to sensitize neurons to degeneration and, accordingly, promoting lipid desaturation within neurons is beneficial for ameliorating C9-associated neurodegeneration in vivo.

### *FAT-1* or *FAT-2* rescue stressor-induced toxicity in C9 iPS cell neurons

**Extended Data Fig. 9:**
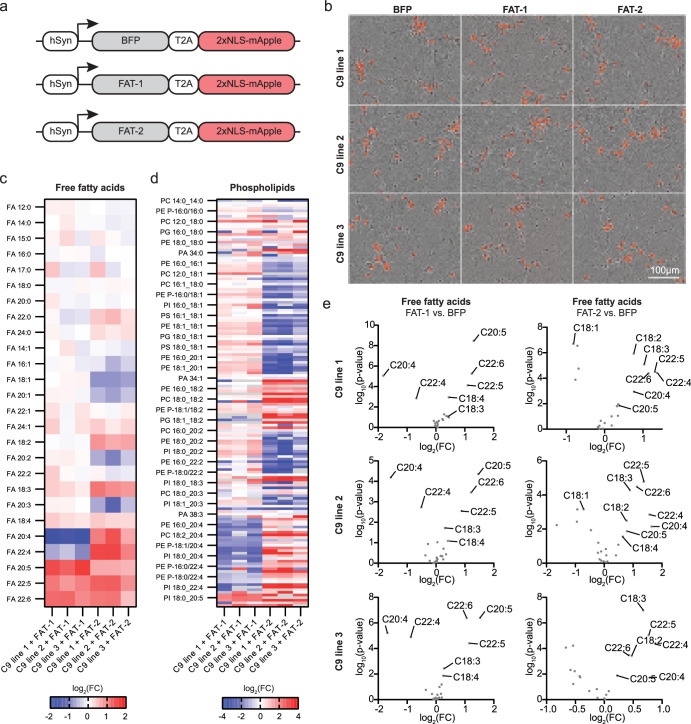
Lipidomic assessment of *FAT-1* and *FAT-2* overexpression in C9 i^3^Neurons. (**a**) Schematic of lentiviral constructs used to overexpress *FAT-1*, *FAT-2*, or a BFP-only control *in vitro*. (**b**) Live-cell images of C9 i^3^Neuron lines overexpressing *BFP*, *FAT-1*, or *FAT-2*, demonstrating high efficiency transduction (as shown by expression of the 2XNLS-mApple reporter). Images representative of at least two inductions per line (n = 3 C9 line 1; n = 2 C9 line 2; n = 3 C9 line 3). (**c, d**) Heatmap displaying all detected (**c**) free fatty acids and (**d**) phospholipids as log_2_(fold-change) over BFP-only control in each C9 line, averaged across three separate neuronal inductions per line. Lipids are normalized by lipid class and sorted by number of double bonds in the most unsaturated fatty acyl chain. Grey boxes indicate lipid species that were either not detected in a condition or are outside the fold-change range. (**e**) Volcano plots of all detected free fatty acid species in *FAT-1* or *FAT-2* overexpression compared to *BFP*-only control. Values represent log_2_(fold-change) over *BFP*-only control and significance (two-sided Student’s t-test) across all replicates/inductions within the labeled group. Source data

We next investigated whether lipid desaturase overexpression can prevent C9-driven neurotoxicity in iPS cell neurons. We first sought to confirm in our i^3^Neuron system that desaturase overexpression is able to increase lipid unsaturation. *FAT-1* is a *C. elegans* lipid desaturase which adds a double bond to ω-6 fatty acids to create the more highly unsaturated ω-3 species^41^. As expected, we observed that lentiviral expression of *FAT-1* caused an increase in ω-3 fatty acids (for example, C20:5 and C22:6) and concomitant decrease in ω-6 species (for example, C20:4), both in the free fatty acid pool and in phospholipids (Extended Data Fig. 9a–e). *FAT-2*, as a Δ12/Δ15 fatty acid desaturase, creates both ω-3 and ω-6 species from less unsaturated precursors^34^. Accordingly, our lipidomic analysis revealed that *FAT-2* overexpression in C9 i^3^Neurons resulted in substantial increases in the essential fatty acids linoleic acid (C18:2) and α-linolenic acid (C18:3), as well as their more highly unsaturated derivatives (for example, C20:4, C20:5 and C22:6), in both the free fatty acid and phospholipid classes (Extended Data Fig. 9a–e). Thus, desaturase overexpression is a potent augmenter of lipid unsaturation in vitro.

**Fig. 5:**
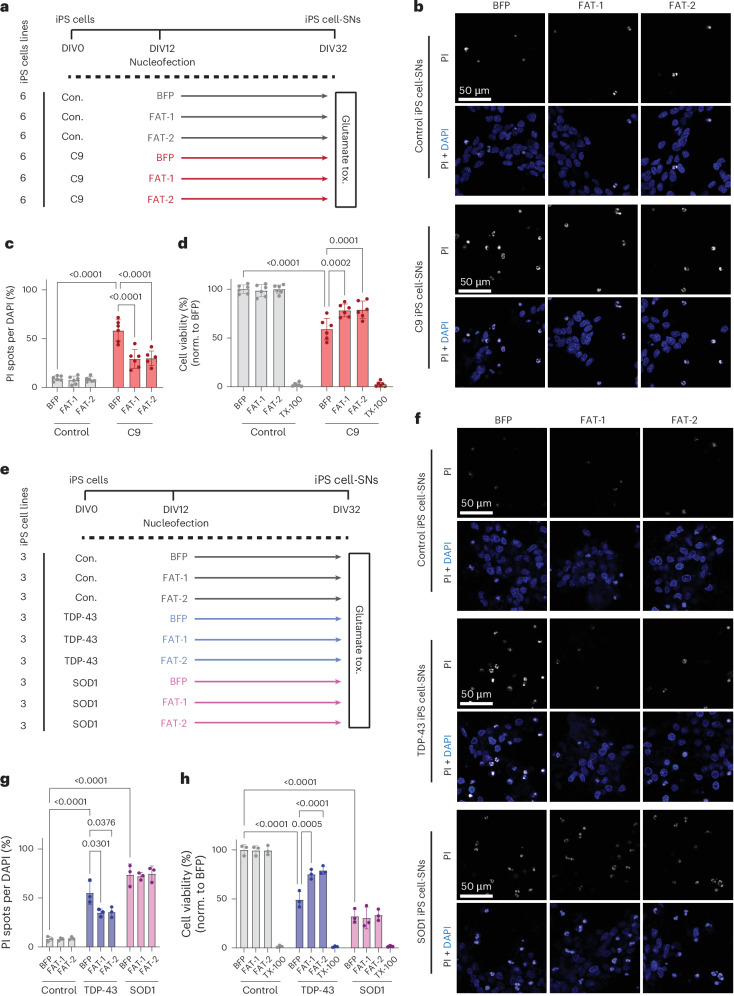
*FAT-1* and *FAT-2* rescue glutamate-induced excitotoxicity in C9 and TDP-43 iPS cell-SNs. **a**, Schematic of SN differentiation timeline, with timepoints of nucleofection and glutamate-induced excitotoxicity measurements. tox., toxicity. **b**, Representative confocal images of cell death in control and *C9orf72* iPS cell-SNs expressing *BFP*, *FAT-1* or *FAT-2*, as measured by PI incorporation. **c**,**d**, Quantification of the ratio of PI-positive (**c**) spots to DAPI-positive (**d**) nuclei (quantification of cell death) and Alamar Blue cell viability assay after 4-h exposure to 10 µM glutamate (*n* = 6 lines per condition). norm., normalized. **e**, Schematic of glutamate-induced excitotoxicity assay in TDP-43 and SOD1 mutant lines. **f**, Representative confocal images of PI incorporation in control, TDP-43 and SOD1 iPS cell-SNs (*n* = 3 lines per condition). **g**,**h**, Quantification of PI incorporation (**g**) and Alamar Blue cell viability assays (**h**) after 4-h exposure to 10 µM glutamate. Datapoints for PI incorporation represent average percentage cell death across ten images per well. Datapoints for Alamar Blue assay represent average percentage viability from three replicate wells for each condition. Two-way ANOVA with Tukey’s multiple-comparison test was used to calculate statistical significance in **c**, **d**, **g** and **h**. Data are presented as mean ± s.d. throughout the figure. Source data

We then tested whether overexpression of desaturase genes could prevent C9-associated neurodegeneration in human C9 neurons. We overexpressed *FAT-1*, *FAT-2* or a *BFP*-only control in C9 iPS-cell-derived spinal neurons (iPS cell-SNs) and then exposed them to high levels of glutamate to induce excitotoxicity (Fig. 5a). As previously reported^10,42^, C9 SNs exhibited heightened susceptibility to excitotoxic cell death compared with SNs derived from healthy donor iPS cells (Fig. 5b–d). Importantly, however, overexpression of either *FAT-1* or *FAT-2* was sufficient to partially rescue glutamate-induced toxicity, significantly decreasing cell death in C9 SNs compared with *BFP*-only control (Fig. 5b–d). Thus, desaturase overexpression is beneficial in preventing C9-associated neurodegenerative phenotypes in human C9 neurons.

Finally, we wondered whether the protective effect of desaturase overexpression might extend to other forms of ALS as well. To test this, we used our glutamate excitotoxicity assay on mutant TDP-43 or SOD1 iPS cell-SNs (Fig. 5e). In both disease groups, we observed heightened vulnerability to glutamate stress compared with control neurons (Fig. 5f–h). Overexpression of either *FAT-1* or *FAT-2* was able to partially rescue toxicity in TDP-43, but not SOD1 neurons (Fig. 5f–h). These results are consistent with the finding that lipid dysregulation is also found in sporadic ALS and FTLD (Fig. 3 and Extended Data Fig. 1c) and suggests that PUFA upregulation may be a common mechanism to combat stress that is protective in non-C9 ALS cases as well.

## Discussion

In the present study, we uncovered lipid metabolism dysregulation in multiple models of C9 ALS/FTD, including transgenic *Drosophila*, iPS-cell-derived neurons and patient postmortem brain and spinal cord tissue of patients with ALS and FTD. We first identified transcriptional dysregulation of canonical fatty acid synthesis and desaturation genes, which was present at a predegeneration timepoint in C9 fly heads and was conserved in end-stage disease in ALS postmortem spinal cord tissue. Through lipidomic assays in C9 flies, iPS-cell-derived neurons and FTLD postmortem brains, we identified a loss of PUFA-containing phospholipids. In vitro, this was recapitulated by transduction of control neurons with 92 G_4_C_2_ repeats and prevented by treatment with a C9 ASO, demonstrating a disease-specific lipid signature. Importantly, promoting lipid desaturation through neuronal desaturase overexpression prolonged survival of C9 flies and rescued glutamate-induced excitotoxicity in C9 and TDP-43 patient iPS cell-SNs. C9 flies also displayed a dramatic defect in cold-stress recovery, a measure of impaired membrane fluidity, which was strongly reversed by neuronal desaturase overexpression. Together, this suggests a functional role for lipid unsaturation in modifying neurodegeneration in ALS/FTD.

Growing evidence links dysregulated lipid homeostasis to neurodegenerative diseases, including ALS/FTD. Several studies have shown altered levels of lipid species in postmortem tissue^43^, cerebrospinal fluid^44,45^ and blood^46–48^ of patients with ALS/FTD as well as ALS rodent models^49^. PUFAs have been specifically linked to ALS pathogenesis, with multiple epidemiological studies suggesting a protective role for dietary PUFAs in decreasing risk of developing ALS^24,25,50^. A recent study of plasma fatty acids from 449 patients with ALS revealed that higher levels of plasma α-linolenic acid (C18:3) at baseline are associated with prolonged survival and slower functional decline, whereas increased plasma linoleic acid (C18:2) and eicosapentaenoic acid (C20:5) were associated with a reduced risk of death during follow-up^23^. Linoleic acid and α-linolenic acid are essential PUFAs that must be obtained from the diet and serve as precursors for the highly unsaturated species arachidonic acid (C20:4), eicosapentaenoic acid (C20:5) and docosahexaenoic acid (C22:6)^51^. In the present study, we report that a wide range of PUFA-containing phospholipids are altered in C9 flies, iPS cell neurons and FTLD postmortem frontal cortex. It is interesting that in our fly experiments the effect appeared to be driven by repeat RNAs, rather than the highly toxic DPRs. This was surprising because the repeat RNA alone is not sufficient to drive neurodegeneration in flies^9,52^. This indicates that the presence of repeat RNA is sensitizing but that additional stressors are required to cause overt neurodegeneration. Consistent with this possibility, knockdown of the fatty acid desaturase *Desat1* in wild-type flies was not sufficient to cause neurodegeneration on its own. This suggests that lipid saturation alteration is probably part of a ‘multistep’ disease process^53^, but importantly it is one that can be modulated to provide benefit. The mechanism by which repeat RNA leads to reduced phospholipid desaturation is not clear, but one possibility is that it could be driven by nucleocytoplasmic transport impairment, which could lead to mislocalization of master transcriptional regulators of lipid metabolism, such as sterol regulatory element-binding proteins or peroxisome proliferator-activated receptors, causing the widespread transcriptional dysregulation of lipid metabolism, with the resulting effects on lipid species reported here^54–56^. Although we see transcriptional changes that probably drive lipid changes in flies, it is possible that there are other triggers in the i^3^Neurons as well as sporadic ALS. For instance, alterations in lipid saturation could be a ‘lipid stress response’ to neurodegenerative insults. Our ‘DPR-only’ flies expressing 36 GR repeats exhibited an almost opposite brain lipid signature to that of RO flies and (G_4_C_2_)_36_ flies, with most PUFA-containing phospholipids appearing as increased compared with controls. As (GR)_36_ flies exhibit a highly aggressive neurodegenerative phenotype, it is still possible that they would also show reduced PUFA-containing phospholipids at earlier timepoints, although at this later disease stage surviving neurons that contain polyunsaturated phospholipids remain. This would be consistent with a loss of PUFAs sensitizing neurons to degeneration. A limitation of our study is the use of one *Drosophila* G_4_C_2_ repeat line, (G_4_C_2_)_36_, preventing us from assessing whether the number of repeats correlates with desaturation levels. Future work comparing lines with varying numbers of G_4_C_2_ repeats, inserted in the same locus, using the same driver and with otherwise genetically identical backgrounds, will help address this interesting question.

Our data fit well with the epidemiological evidence of PUFA levels and intake being protective in ALS, but crucially suggest that delivery of PUFAs to neurons is a key determinant of their protective function. We were able to study C9 lipid dysregulation specifically in neurons by using an inducible neuronal driver in *Drosophila* and employing pure neuronal cultures for lipidomic analyses. Using this approach, we observed a strikingly enhanced benefit of neuronal overexpression of desaturases in flies versus feeding PUFAs in the diet. To reach the brain, PUFAs need to pass the gut barrier, as well as the blood–brain barrier (BBB), and therefore the absolute quantities that reach neurons from the diet are unclear. A metabolic labeling study recently suggested that dietary sources account for 60–70% of the PUFAs in the mouse brain^57^. However, the efficiency of BBB transport varies for each fatty acid species^58^. This delivery issue may explain the differences in survival benefits observed between genetic overexpression of desaturases versus pharmacological supplementation of their fatty acid products, although we cannot rule out that dietary linoleic acid and α-linolenic acid may partially mediate their survival benefits through systemic actions. Furthermore, we have been able to show increased neuronal protection with increased unsaturation, demonstrating that any single gene in the lipid synthesis and desaturation pathway that can ultimately increase PUFAs in neurons can be beneficial; however, the extent of benefit is greater as one goes further down the pathway and increases the degree of unsaturation, with the benefit greatest with *FAT-2*, then *Desat1*, followed by *FASN1* and *FASN2*.

Aging is a major risk factor for both ALS and FTD^59,60^. It is interesting that overexpression of either *FASN1* or *Desat1* in neurons also significantly increased wild-type fly lifespan (although to a lesser degree than in C9 flies), suggesting that this pathway may also be beneficial to aging neurons and warrants further investigation in this context. Furthermore, we observed lipid-related transcriptional dysregulation and decreased PUFA-containing phospholipids, not only in our C9 models but also in non-C9 ALS/FTD postmortem material. Although our data show that C9 repeats are sufficient to drive lipid saturation changes, there must be other parallel pathways that induce these changes in sporadic forms of the disease and aging-related changes are an obvious candidate. Taken together, impaired lipid metabolism is a common dysregulated pathway in ALS/FTD and it will now be important to investigate the different drivers of lipid-related changes.

Lipid saturation, along with fatty acyl chain length and head group composition, influences membrane physicochemical properties and physiological functions^61,62^. The role of unsaturated lipids in modulating membrane fluidity has been well described^36,63–67^. Our study explored membrane fluidity in a physiological paradigm by testing the ability of flies to recover from cold stress. Flies neuronally expressing (G_4_C_2_)_36_ were sensitive to cold stress, which was ameliorated by overexpressing either *Desat1* or *FAT-2* with the same neuronal driver, suggesting that these desaturase enzymes are fluidizing neuronal membranes. Future studies are now warranted to assess the role of neuronal membrane fluidity in neurodegeneration. In addition to altering membrane dynamics, other mechanisms may also be involved. For example, PUFAs can be de-esterified from membrane phospholipids and converted to bioactive signaling molecules known as oxylipins^68–71^. Elevated levels of arachidonic acid-derived oxylipins, called eicosanoids, have previously been reported in ALS motor neurons, whereas inhibiting their production through 5-LOX inhibition has been shown to rescue toxicity in the developing eye in a C9 *Drosophila* model^31^, thus highlighting another PUFA-related pathway that may contribute to disease.

Although we focus here on neuronal lipids, future work may benefit from expanding these studies to glial and co-culture paradigms to unpick the interplay between different cell types. Indeed, recent work demonstrated that reactive astrocytes secrete saturated fatty acids, which promote motor neuron degeneration in ALS models^72–75^, whereas astrocyte-specific knockout of *ELOVL1*, an enzyme responsible for producing long-chain saturated lipids, reduced astrocyte-mediated neuronal toxicity in vitro and in vivo^72^. These data are in line with our findings because they converge on the hypothesis that PUFAs are protective to neurons whereas saturated fatty acids are harmful, which further highlights an important role for lipid desaturation in ALS/FTD pathogenesis. Overall, the results presented in the present study identify dysregulated lipid metabolism as a direct contributor to neuronal toxicity in C9 ALS/FTD and suggest that modulating neuronal lipid saturation is a promising approach for ameliorating neurodegeneration.

## Methods

### Ethics statement

Patient iPS cell lines were collected with prior informed patient consent and derived from biopsied fibroblasts. Ethical approval was received from the National Healtlh Service (NHS) Health Research Authority East of England, Essex Research Ethics Committee (REC, reference no. 18/EE/0293). Brains were donated to the Queen Square Brain Bank (QSBB; UCL Queen Square Institute of Neurology) with full informed consent. Clinical and demographic data for all brains used in the present study were stored electronically in compliance with the 1998 Data Protection Act and are summarized in Supplementary Table 1. Ethical approval for the study was obtained from the NHS research ethics committee and in accordance with the human tissue authority’s code of practice and standards under license no. 12198. All cases underwent a pathological diagnosis for FTLD according to current consensus criteria^76,77^.

### *Drosophila* maintenance

*Drosophila* stocks were maintained on SYA food (15 g l^−1^ of agar, 50 g l^−1^ of sugar, 100 g l^−1^ of autolysed yeast, 30 ml l^−1^ of nipagin (10% in ethanol) and 3 ml l^−1^ of propionic acid) at 25 °C in a 12-h light:dark cycle with 60% constant humidity. For RU486-induced experiments, food was supplemented with 200 µM RU486 (mifepristone). The elavGS stock was derived from the original elavGS 301.2 line^78^ and generously provided by H. Tricoire (CNRS, France)^79^. UAS-FASN1 and UAS-FASN2 lines were a gift from J. Montagne (Université Paris-Sud)^80^. The UAS-FASN RNAi line was obtained from Vienna *Drosophila* Resource Center (VDRC, cat. no. v29349). The w1118 line (BDSC, cat. no. 3605), GMR-GAL4 line (BDSC, cat. no. 9146) and UAS-Desat1 RNAi line (BDSC, cat. no. 37512) were obtained from the Bloomington Drosophila Stock Center (BDSC). The UAS-Desat1 (DGRC, cat. no. 118679), UAS-FAT-2 (DGRC, cat. no. 118682) and UAS-Desat1(42) (DGRC, cat. no. 118681) lines were obtained from the Kyoto Drosophila Stock Center^33^. The UAS-(G_4_C_2_)_36_, RO and (GR)_36_ stocks have been previously described^9,27^. All stocks were backcrossed to either a w1118 strain or v-w1118 stock for six generations before use in experiments. Stocks used in the present study are listed in Supplementary Table 2.

### Fatty acid supplementation to *Drosophila* food

Fatty acids were added to SYA food, along with 200 µM RU486 (Sigma-Aldrich), although it was still liquid but had cooled to 50 °C. The food was mixed thoroughly with an electric handheld blender, before dispensing into individual vials. Fatty acids used were palmitic acid (Merk, cat. no. W283215), stearic acid (Thermo Fisher Scientific, cat. no. 10002390), oleic acid (Merck, cat. no. W281506), linoleic acid (Merck, cat. no. 436305) and α-linolenic acid (Merck, cat. no. L2376).

### *Drosophila* behavioral and lifespan assays

#### Lifespan assays

The parental generation of experimental crosses was allowed to lay for 24 h on grape-agar plates supplemented with yeast paste. Eggs were washed briefly in 1× phosphate-buffered saline (PBS), pH 7.4 before being dispensed into bottles using a pipette at a standard density (20 µl of eggs in PBS, approximately 300 eggs). Then, 2-d post-eclosion flies were allocated to experimental vials at a density of 15 flies per vial. Deaths were scored and flies tipped on to fresh food at least 3× a week. All lifespans were performed at 25 °C on mated females.

#### *Drosophila* eye phenotype analysis

Flies carrying the UAS-FASN1 RNA interference (RNAi) or UAS-Desat RNAi construct were crossed to GMR-GAL4 flies at 25 °C. Then 2-day-old adult F1 female flies were used, with one eye per fly imaged using a stereomicroscope. All images were obtained under the same magnification; eye area was calculated from each image using Fiji.

#### Assessment of *Drosophila* feeding

The 2-day-old mated female flies were transferred to SYA food containing 200 μM RU486 or ethanol vehicle control with PUFAs at a density of five per vial on the evening before the assay, with between seven and nine replicate vials per experimental group. Vials were coded and placed in a randomized order in rows on viewing racks at 25 °C overnight. The next day, observations were performed ‘blind’ for 90 min, commencing 1 h after lights on and 30 min after the arrival of the observer to the room. In turn, each vial was observed for approximately 5 s, during which the number of flies feeding was noted. A feeding event was scored when a fly had its proboscis extended and touching the food surface while performing a bobbing motion. Once all vials in the experiment had been scored, nine additional rounds of observations were carried out in the same way for the whole 90 min. At the end of the assay, the vial labels were decoded and the feeding data expressed as a proportion by experimental group (sum of scored feeding events divided by total number of feeding opportunities, where total number of feeding opportunities = no. of flies in vial × no. of vials in the group × no. of observations)^81^. For statistical analyses, comparisons between experimental groups were made on the totals of feeding events by all flies within a vial, to avoid pseudoreplication.

#### Cold-stress recovery assay

*Drosophila* were induced on SYA medium containing 200 µM RU486 or ethanol vehicle control for 7 d, before exposure to 4 °C for 18 h to cause a cold-induced paralysis response. At the end of this period, lies were moved to room temperature for 1 h and recovery was assessed^39^. The number of flies exhibiting a full recovery (walking), partial recovery (partial paralysis) or death were quantified and expressed as a percentage of the total. The results were analyzed using the *χ*^2^ test.

### *Drosophila* RNA-seq

Adult female flies were induced on SYA medium containing 200 µM RU486 or ethanol vehicle control for 5 d and subsequently snap frozen. Total RNA was isolated from 15 heads per replicate using TRIzol, and the experiment was performed in quadruplicate. RNA-seq was performed with an Illumina NextSeq2000, using 16 million paired-end reads per sample and 100-bp read length. Raw sequence reads were aligned to the Dm6 reference genome. DESeq2 (default parameters) was used to perform differential expression analysis (DEGs provided in Supplementary File 2). The ‘runTest’ function from the topGO package (v.2.53.0)^82^ was used to perform GO enrichment analysis on DEGs (log_2_(fold-change) > 0.58). The ‘weight01’ algorithm and ‘fisher’ statistic were used when running topGO. The ‘GenTable’ function was used to generate a table with the top biological process GO terms. Plots with topGO terms were plotted using ggplot2 (v.3.4.2). We generated a heatmap for topGO terms showing the percentage of significant DEGs among all genes of a GO term expressed in a dataset using the pheatmap function from the pheatmap package (v.1.0.12, https://CRAN.R-project.org/package=pheatmap).

### RT–qPCR

Total RNA from fly heads was extracted from 15 heads per replicate, as above. Total RNA from i^3^Neurons was extracted from one well of a six-well plate per technical replicate using the using the Promega ReliaPrep RNA Cell Miniprep System using the manufacturer’s protocol, including DNase I digestion.

For reverse transcription (RT) in fly head samples, approximately 1 μg of RNA per sample (10.6 µl) was incubated with 2 µl of TURBO DNase (Thermo Fisher Scientific) and 1.4 µl of TURBO DNase buffer (Thermo Fisher Scientific) at 37 °C for 15 min. After this, the reaction was inhibited with addition of 2 µl of EDTA to a final concentration of 3.4 mM, followed by incubation at 75 °C for 5 min. Then 2 µl of 0.5 µg µl^−1^ of oligo dT and 2 µl of dNTP mix (10 mM stock made from individual 100 mM dNTP stocks, Invitrogen) were added to each sample followed by a 5-min incubation at 65 °C. After this, samples were placed on ice. To each reaction, the following was added: 8 µl of 5× first-strand buffer, 8 µl of 25 mM MgCl_2_, 4 µl of 0.1 M dithiothreitol, 2 µl of RNaseOut RNase inhibitor (40 units µl^−1^) and 1 µl of SuperScript II reverse transcriptase (Invitrogen). Samples were incubated at 42 °C for 50 min, then heat inactivated at 70 °C for 15 min. Quantitative (q)PCR was performed using the QuantStudio 6 Flex Real-Time PCR System (Applied Biosystems) using SYBR Green master mix (Applied Biosystems). Relative messenger RNA levels were calculated relative to αTub84B expression by the comparative *C*^t^ method. Primer sequences used are described in Supplementary Table 3.

Reverse transcription from i^3^Neuron samples was carried out with SuperScript IV Vilo (Thermo Fisher Scientific), using the manufacturer’s protocol and 75 ng of total RNA per technical replicate. Then qPCR was performed with the LightCycler 480 and SYBR Green master mix. Relative mRNA levels were calculated relative to glyceraldehyde 3-phosphate dehydrogenase (GAPDH) expression using the comparative *C*^t^ method. All primer pairs were from the predesigned catalog from IDT and were confirmed to have 90–110% efficiency in our hands with i^3^Neuron complementary DNA: GAPDH (Hs.PT.39a.22214836), FASN (Hs.PT.58.38567473), SCD (Hs.PT.58.45714389), SCD5 (Hs.PT.58.40730206), FADS1 (Hs.PT.4716384) and FADS2 (Hs.58.15091651).

### DPR MSD immunoassays

#### *Drosophila* head protein preparation

Adult female flies were induced on SYA medium containing 200 µM RU486 or ethanol vehicle control for 7 d and subsequently 10 heads per sample were homogenized in 100 μl of 2% sodium dodecylsulfate (SDS) buffer (Merck, cat. no. 428018) containing 1× radioimmunoprecipitation (RIPA) buffer (Sigma-Aldrich, cat. no. R0278) and complete mini EDTA-free protease inhibitor cocktail (Roche, cat. no. 11836170001) at room temperature for 30 s until the heads were no longer intact. Samples were then heated at 95 °C for 10 min. After centrifugation at 18,400*g* for 20 min at room temperature, the supernatants were collected in the new tubes. The protein concentration was determined using Pierce BCA Protein Assay Kit (Thermo Fisher Scientific, cat. no. 23325) according to the manufacturer’s manual.

#### Protein preparation of i^3^Neurons

The i^3^Neuron replicates for DPR Meso Scale Discovery (MSD) were collected alongside those used for lipidomic analyses from the same neuronal inductions. One well of a six-well plate was used per replicate for MSD. At DIV21, neurons were lifted with PBS, centrifuged and pelleted at 1,500*g* for 5–10 min, snap frozen on dry ice and stored at −80 °C until use. For protein preparation, cell pellets were resuspended in 200 μl of 2% SDS buffer (Thermo Fisher Scientific, cat. no. BP2436-200) containing 1× RIPA buffer and cOmplete mini EDTA-free protease inhibitor cocktail and sonicated 2× for 10 s at 30 A and 4 °C. Sonicated samples were centrifuged at 17,000*g* for 20 min at 16 °C, after which supernatants were collected and used in MSD assays.

#### Running MSD assays

Samples were diluted to the same concentration with homogenization buffer and 25 μl (fly samples) or 90 µl (cell samples) was loaded in duplicate on to the 96-well MSD immunoassay plate. Singleplex MSD immunoassays to measure poly(GA) or poly(GP) levels were previously validated^83^. The following antibodies were used: anti-poly(GP) (GP658, custom-made from Eurogentec, 2 µg ml^−1^) and anti-poly(GA) (Merck Millipore, clone 5E9, cat. no. MABN889, 1 µg ml^−1^) as capture antibodies, and biotinylated anti-poly(GP) (GP658*, 1 µg ml^−1^) and biotinylated anti-poly(GA) (GA5F2*, kindly provided by D. Edbauer (Ludwig-Maximilians-Universität, Munich), biotinylated in house, 1 µg ml^−1^) as detector antibodies. Plates were read with the MSD reading buffer (cat. no. R92TC) using the MSD Sector Imager 2400. A four-parameter logistic regression curve was fit to the values obtained from a standard curve using GraphPad Prism and concentrations were interpolated. Signals correspond to the intensity of emitted light on electrochemical stimulation of the assay plate. Before analysis, the average reading from a calibrator containing no peptide was subtracted from each reading.

### Differentiation of i^3^Neurons

C9 patient and isogenic control iPS cell lines were kind gifts of the Chandran laboratory at the University of Edinburgh^42^ and C9 repeat knock-in lines on the KOLF2.1J background were a gift from the Skarnes laboratory at Jackson Labs as part of the iPS cell Neurodegenerative Disease Initiative^84,85^ (line details in Supplementary Table 4). From these, we generated i^3^-compatible iPS cell lines via piggyBac-integration of a BFP-containing, doxycycline-inducible *Neurogenin2* (*Ngn2*) minigene (kind gift of M. Ward, NIH). After integration, iPS cells were subsequently doubly selected with puromycin and FACS, resulting in a pure population of stably expressing iPS cells. These i^3^iPS cells were then used for rapid differentiation into cortical neurons (i^3^Neurons) using a previously described method^28,29^. Briefly, i^3^iPS cells were grown to 70–80% confluency, washed with PBS, lifted with Accutase (Gibco) and plated at 375,000 cells per well in a 6-well plate on to Geltrex-coated plates (DIV0). Cells were maintained from DIV0–3 in an induction medium consisting of Dulbecco’s modified Eagle’s medium (DMEM-F12; Gibco), 1× N2 (Thermo Fisher Scientific), 1× Glutamax (Gibco), 1× Hepes (Gibco), 1× nonessential amino acids (Gibco), doxycycline (2 µg ml^−1^) and 10 µM Y-27632 (DIV0 only; Tocris), which was exchanged daily. On DIV3, cells were dissociated with accutase and replated on to poly(l-ornithine)- (Merck) or poly(ethylenimine)- (Sigma-Aldrich) and laminin- (Sigma-Aldrich) coated 6-well plates at 600,000 cells per well in neuronal maintenance medium consisting of Neurobasal medium (Gibco), supplemented with 1× B27 (Gibco), 10 ng ml^−1^ of brain-derived neurotrophic factor (PeproTech), 10 ng ml^−1^ of NT-3 (PeproTech) and 1 µg ml^−1^ of laminin. From DIV3 to DIV21, cells were maintained in neuronal maintenance medium, with one-third medium changes once weekly. Lentiviral transduction to overexpress (G_4_C_2_)_92_ or (G_4_C_2_)_2_ was done 1 h after DIV3 replating. Likewise, ASO treatments to target the *C9orf72* sense strand or an NT control were also begun on DIV3 and supplemented in medium changes thereafter. In brief, 1 h after replating, ASOs were transiently transfected using Lipofectamine Stem (Invitrogen, cat. no. STEM00015) at 5 μM final concentration according to the manufacturer’s protocol. Then 1 d after ASO treatment, a full medium change was done to remove remaining Lipofectamine Stem and replaced with neuronal maintenance medium containing 5 μM ASO, which was then further re-supplemented in weekly medium changes at 5 μM. ASOs were published in ref. ^7^ and have fully modified phosphorothioate backbones. The sequences are as follows, with the five 2ʹ-*O*-methyl RNA base-pairs on either end (italicized):

C9 sense targeting: *UACAG*GCTGCGGTTG*UUUCC*

NT: *CCUUC*CCTGAAGGTT*CCUCC*

### (G_4_C_2_)_92_ or (G_4_C_2_)_2_ lentiviral construct subcloning

The pCDH-EF1-MCS-IRES-copGFP lentiviral plasmid (System Biosciences) was used as the backbone to create (G_4_C_2_)_92_ and (G_4_C_2_)_2_ lentiviral constructs. Subcloning to insert the repeats was undertaken in a two-step process. First, we synthesized a DNA fragment consisting of a customized multiple cloning site (MCS) sandwiched in between 300 bp each of repeat-adjacent upstream and downstream sequences from *C9orf72* intron 1 and then inserted into the internal MCS of pCDH-EF1-MCS-IRES-copGFP with InFusion cloning (Takara Bio) in between XbaI and NotI restriction sites. This interim construct, termed ‘pCDH-EF1-C9up-MCS-C9down-IRES-copGFP’ was verified with diagnostic restriction digests and Sanger sequencing across the insert. Then, to create the (G_4_C_2_)_92_ construct, a 92-repeat sequence was isolated from a previously verified in-house construct with NheI and NotI restriction digests and subcloned into the MCS of pCDH-EF1-C9up-MCS-C9down-IRES-copGFP with overnight ligation at 4 °C (T4 ligase, NEB). To maintain repeat stability, bacterial clones were grown at room temperature, in half the standard ampicillin concentration (0.5 mg ml^−1^), and in low-salt lysogeny broth (Sigma-Aldrich). A repeat-stable clone was selected and subsequently maxi-prepped (QIAGEN) for use in lentiviral production. Thus, the final construct consisted of 92 repeats immediately surrounded on either side by 300 bp of endogenous *C9orf72* intronic sequence to facilitate RAN translation and upstream of an IRES-copGFP sequence for fluorescent visualization of transduction efficiency. To create the (G_4_C_2_)_2_ control lentiviral constructs, two complementary short oligos were synthesized containing two G_4_C_2_ repeats and NheI and NotI restriction site overhangs. Oligos were resuspended in annealing buffer (NEB buffer 2.1), heated to 95 °C and allowed to cool slowly to room temperature to anneal. Annealed oligos were used directly in ligation reactions into pCDH-EF1-C9up-MCS-C9down-IRES-copGFP with the same protocol as used for the 92-repeat construct.

### Lentiviral production

HEK293T cells were grown at 37 °C and 5% CO_2_ in T175 flasks. At ~70% confluency, cells were transfected with either (G_4_C_2_)_92_ or (G_4_C_2_)_2_ lentiviral transfer plasmids along with PAX (Addgene, cat. no. 12260) and vesicular stomatitis virus G (Addgene, cat. no. 12259) lentiviral packaging plasmids with Lipofectamine 3000 Transfection Reagent (Invitrogen) according to the manufacturer’s protocol. After 48 h, medium was collected and centrifuged at 500*g* for 10 min at 4 °C to remove cell debris, after which Lenti-X Concentrator (Takara Bio) was added at a 1:3 ratio. After a minimum incubation of 24 h at 4 °C, the concentrator-medium mix was centrifuged at 1,500*g* and 4 °C for 45 min and the resulting concentrated lentiviral pellet was resuspended in sterile PBS, aliquoted and stored at −80 °C until use.

### Targeted lipidomics of *Drosophila* brains, human i^3^Neurons and postmortem brain samples

#### Sample collection

Adult female flies were induced on SYA medium containing 200 µM RU486 or ethanol vehicle control for 7 d. 60 flies per condition were dissected in PBS on ice, with 20 brains per biological replicate. The brains were collected in 300 µl of PBS and centrifuged at 375*g* for 5 min at 4 °C. Then, 200 µl of supernatant was removed and samples were homogenized before fast-freezing on dry ice. Samples were stored at −80 °C until analysis. For i^3^Neurons, at DIV21 cells were pelleted and stored at −80 °C until analysis. Postmortem brain samples were from pathologically diagnosed cases of FTLD without *C9orf72* expansion (*n* = 32), FTLD with *C9orf72* expansion (*n* = 15) and neurologically normal controls (*n* = 13). One sample from a non-C9 FTLD patient was included twice as a technical replicate. Frontal cortex gray matter and cerebellum were dissected from each brain and stored at −80 °C until analysis.

#### Targeted lipidomic measurements

Comprehensive targeted lipidomics was accomplished using a flow-injection assay based on lipid class separation by differential mobility spectroscopy and selective multiple reaction monitoring (MRM) per lipid species (Lipidyzer platform, SCIEX). A very detailed description of lipid extraction, software and the quantitative nature of the approach can be found elsewhere^86–88^. In short, after the addition of >60 deuterated internal standards (ISs), lipids were extracted using methyl *tert*-butyl ether. Organic extracts were combined, dried under a gentle stream of nitrogen and reconstituted in running buffer. Lipids were then analyzed using flow injection in MRM mode employing a Shimadzu Nexera series HPLC and a Sciex QTrap 5500 mass spectrometer. For the internal calibration, deuterated IS lipids for each lipid class were used within the lipidomics workflow manager. Each lipid species was corrected by the closest deuterated IS within its lipid class and afterwards the obtained area ratio was multiplied by the concentration of the IS.

### Analyses of targeted lipidomic datasets

#### Filtering and normalizations

Raw amounts of individual lipid species were obtained from the Lipidyzer platform as above and subsequently filtered and normalized. Datasets were first filtered for low-abundance and undetected lipid species. To pass filtering, a lipid species must be detected in at least 80% of all samples in the analysis or in 60% of samples in any one group, and must also be at least twofold above the average of the blanks. After filtering, missing sample values were imputed as the median of other samples in their group; this step was found to be necessary for subsequent normalizations, because missing values greatly skewed the proportional datasets. Next, filtered and imputed datasets were normalized either to total lipids (for analysis of class-level lipid alterations, as in Extended Data Figs. 5a and 6c,d) or by lipid class individually (as in Figs. 2b,c and 3b,c and Extended Data Fig. 3a–e). Thus, these processing steps result in proportional lipidomic measurements, relative to either the total lipidome or total amount of lipid within each class, respectively.

#### Fold-changes in *Drosophila* brains

Biological replicates for these analyses contained 20 fly brains per condition. Each replicate was filtered and normalized individually by lipid class and then fold-changes and significance were calculated as the average of either C9/RO/GR36 over the average of the control (w1118) or (G_4_C_2_)_36_ + Desat1 /(G_4_C_2_)_36_ + FAT-2 over the average of (G_4_C_2_)_36_, using lipid class-normalized data.

#### Fold-changes in i^3^Neurons

For comparisons of C9 lines to their isogenic controls, we defined biological replicates as individual iPS cell lines and employed three separate C9 lines for lipidomic analysis. Technical replicates were defined as individual wells that were grown, collected and analyzed separately. To ensure reproducibility, technical replicates were collected across multiple neuronal inductions, and each lipidomic dataset was normalized to the average of the control condition within induction. For our main analysis, we compared each line with its own isogenic control, using technical replicates across inductions for statistical analysis and displayed the results for each individual line separately (Fig. 2c and Extended Data Fig. 3b,c). To demonstrate reproducibility across inductions, we also show fold-change from each individual neuronal induction separately in Extended Data Fig. 3a. The same rationale and analysis were applied to the ASO experiments, but rather comparing C9 ASO-treated lines with their own NT ASO-treated conditions, again with normalizations within induction (Fig. 2c and Extended Data Fig. 3d,e). For the 92-repeat lentiviral experiments, because this is an exogenous overexpression paradigm, we instead defined biological replicate units as individual neuronal inductions and performed three separate inductions for lipidomic analyses. Two of these inductions were done with control line 1 and the other with control line 2. For our main analysis in Fig. 2c, we then combined and compared technical replicates across the three inductions between 92-repeat and 2-repeat treated conditions; however, to demonstrate reproducibility across inductions, we also show fold-changes from each neuronal induction separately in Extended Data Fig. 3a. All i^3^Neuron lipidomic datasets are publicly available in user-friendly format at https://neurolipidatlas.nl, where any user can view and analyze each experiment, with full statistical analysis, either by induction separately or by all technical replicates combined by line or treatment condition.

#### Fold-changes and unsaturation indices in postmortem brain samples

Fold-changes in FTLD versus control samples, as used in the heatmap in Fig. 3b, were calculated for each lipid species separately as the average of the FTLD condition over the average of the control condition, using lipid class-normalized data. To calculate the unsaturation index in Fig. 3c, a composite score was calculated for each sample individually, using the ratio of the sum of phospholipid species with four to six double bonds in their most highly unsaturated fatty acyl chain over the sum of species with zero to three double bonds, after lipid class normalization. Each individual was considered a separate biological replicate and two-way analysis of variance (ANOVA) was used to calculate statistical significance across the two brain regions and between the FTLD and control. Normality tests were performed for each group with D’Agostino and Pearson’s test which determined each group to be normally distributed (*α* = 0.05).

### Glutamate-induced excitotoxicity assays in iPS cell-SNs

#### Subcloning for *BFP*, *FAT-1* and *FAT-2* overexpression constructs

The *mTagBFP* (Addgene, cat. no. 89685), *FAT-1* (Genscript) and *FAT-2* (Genscript) cDNAs were amplified with PCR and subcloned into the pHR-hSyn-eGFP vector (Addgene, cat. no. 114215), along with a T2A-NLS-mApple minigene for fluorescent visualization. In brief, enhanced green fluorescent protein (eGFP) was removed with BamHI and NotI (NEB) and *BFP/FAT-1/FAT-2* and T2A-NLS-mApple fragments were inserted with InFusion cloning (Takara Bio), as per the manufacturer’s protocol. The resulting plasmids were verified with diagnostic restriction digest and Sanger sequencing before being maxi-prepped (QIAGEN) for subsequent use in excitotoxicity assays.

#### Excitotoxicity assays

Non-neurological control and *C9orf72* iPS cells were obtained from the Answer ALS repository at Cedars Sinai (see Supplementary Table 5 for demographics) and maintained in mTeSR Plus medium at 37 °C with 5% CO_2_. The iPS cell-SNs were differentiated according to a modified diMNs protocol^56,89–91^ and maintained at 37 °C with 5% CO_2_. The iPS cells and iPS cell-SNs were routinely tested negative for *Mycoplasma*. On day 12 of differentiation, iPS cell-SNs were dissociated with trypsin; 5 × 10^6^ iPS cell-SNs were nucleofected with 4 µg of plasmid DNA in suspension. After nucleofection, 100 µl of cell suspension was plated in each well (total of 6 wells per cuvette) of a glass-bottomed or plastic 24-well plate for propidium iodide (PI) and Alamar Blue toxicity and viability experiments, respectively. Medium was exchanged daily for a total of 20 d to facilitate the removal of iPS cell-SNs that failed to recover post-nucleofection. On the day of the experiment (day 32 of differentiation), iPS cell-SN medium was replaced with artificial cerebrospinal fluid solution containing 10 µM glutamate. For those iPS cell-SNs undergoing Alamar Blue viability assays (plastic dishes), Alamar Blue reagent was additionally added to each well according to the manufacturer’s protocol at this time. After incubation, iPS cell-SNs for PI cell death assays were incubated with PI and NucBlue live ready probes for 30 min and subjected to confocal imaging. The number of PI spots and nuclei were automatically counted in Fiji. Alamar Blue cell viability plates were processed according to the manufacturer’s protocol. As a positive control, 10% Triton X-100 was added to respective wells 1 h before processing.

### Statistics and reproducibility

The statistical test used for each experiment is indicated in the figure legends. The log-rank tests for fly survival were performed in Microsoft Excel (template described in ref. ^92^). ANOVA or Student’s *t*-test analyses were performed in GraphPad Prism v.10.0.2. For all statistical tests, *P* < 0.05 was considered significant. Data distribution was tested for normality only where specifically stated in Methods, otherwise data distribution was assumed to be normal, but this was not formally tested. No statistical methods were used to predetermine sample sizes but our sample sizes are similar to those reported in previous publications^9,52,90^ and are listed in the figure legends for each experiment. For fly survival assays, roughly *n* = 150 flies were used per condition. For iPS cell-neuron experiments, the number of lines used is listed in Supplementary Tables 4 and 5 and, for lipidomics, the individual inductions are shown separately in Extended Data Fig. 3a. For human postmortem brain lipidomic experiments, there were *n* = 13 control and *n* = 45 FTLD frontal cortex samples, and *n* = 13 control and *n* = 47 FTLD cerebellum samples. Experimental groups were determined by genetic status and not randomized. Data collection and analysis were not performed blind to the conditions of the experiments unless specifically stated in Methods. No datapoints were excluded from the analyses.

### Reporting summary

Further information on research design is available in the Nature Portfolio Reporting Summary linked to this article.

## Online content

Any methods, additional references, Nature Portfolio reporting summaries, source data, extended data, supplementary information, acknowledgements, peer review information; details of author contributions and competing interests; and statements of data and code availability are available at 10.1038/s41593-025-01889-3.

## Supplementary information

Supplementary Information Supplementary Tables 1–5.

Reporting Summary

## Source data

Source Data Fig. 1 Statistical source data.

Source Data Fig. 2 Statistical source data.

Source Data Fig. 3 Statistical source data.

Source Data Fig. 4 Statistical source data.

Source Data Fig. 5 Statistical source data.

Source Data Extended Data Fig. 1 Statistical source data.

Source Data Extended Data Fig. 2 Statistical source data.

Source Data Extended Data Fig. 3 Statistical source data.

Source Data Extended Data Fig. 4 Statistical source data.

Source Data Extended Data Fig. 5 Statistical source data.

Source Data Extended Data Fig. 6 Statistical source data.

Source Data Extended Data Fig. 7 Statistical source data.

Source Data Extended Data Fig. 8 Statistical source data.

Source Data Extended Data Fig. 9 Statistical source data.

## Data Availability

All transcriptomic data generated in the present study are deposited in the Genome Expression Omnibus, accession no. GSE255099. All lipidomic data generated from *Drosophila* brains, i^3^Neurons and postmortem brains are publicly available for user-friendly exploration in the recently described Neurolipid Atlas (https://neurolipidatlas.nl) and can be found by selecting ‘Isaacs’ as the contributing lab^93^. Source data are provided with this paper.
