# Author Correction: Neuronal polyunsaturated fatty acids are protective in ALS/FTD

**DOI:** 10.1038/s41593-025-01926-1

**Published:** 2025-03-07

**Authors:** Ashling Giblin, Alexander J. Cammack, Niek Blomberg, Sharifah Anoar, Alla Mikheenko, Mireia Carcolé, Magda L. Atilano, Alex Hull, Dunxin Shen, Xiaoya Wei, Rachel Coneys, Lele Zhou, Yassene Mohammed, Damien Olivier-Jimenez, Lian Y. Wang, Kerri J. Kinghorn, Teresa Niccoli, Alyssa N. Coyne, Rik van der Kant, Tammaryn Lashley, Martin Giera, Linda Partridge, Adrian M. Isaacs

**Affiliations:** 1 UK Dementia Research Institute, UCL, London, UK; 2 Institute of Healthy Ageing, UCL, London, UK; 3 Department of Neurodegenerative Disease, UCL Queen Square Institute of Neurology, London, UK; 4 Center for Proteomics & Metabolomics, Leiden University Medical Center, Leiden, The Netherlands; 5 Department of Neurology, Johns Hopkins University, Baltimore, MA USA; 6 Brain Science Institute, Johns Hopkins University, Baltimore, MA USA; 7 Alzheimer Center Amsterdam, Amsterdam University Medical Center, Amsterdam, The Netherlands; 8 Present Address: MRC Laboratory of Molecular Biology, Cambridge, UK

**Keywords:** Amyotrophic lateral sclerosis, Molecular neuroscience

Correction to: *Nature Neuroscience* 10.1038/s41593-025-01889-3, published online 25 February 2025.

In the version of the article initially published, Martin Giera was listed with the wrong affiliation which has now been amended to the Center for Proteomics & Metabolomics, Leiden University Medical Center, Leiden, The Netherlands in the HTML and PDF versions of the article.

