# Supplementary material for: Neuronal polyunsaturated fatty acids are protective in ALS/FTD
Source: Nat Neurosci. 2025 Feb 25;28(4):737–47. doi: 10.1038/s41593-025-01889-3 (PMC11976277; doi:10.1038/s41593-025-01889-3)
Supplement: Supplementary file 1 — Supplementary Tables 1–5. [file 41593_2025_1889_MOESM1_ESM.pdf]

nature neuroscience

Supplementary information https://doi.org/10.1038/s41593-025-01889-3

Neuronal polyunsaturated fatty acids are protective in ALS/FTD

In the format provided by the authors and unedited

Supplementary Information

Supplementary Table 1. Clinical and demographic data for post-mortem brain samples

Group	Sample size	Age at onset (Avg. +/- SD)	Age at death (Avg. +/- SD)	% Male	Pathological Diagnosis	Mutations
Control (Frontal cortex)	n=13	N/A	74 ± 6.1	53.8%	N/A	N/A
Control (Cerebellum)	n=13	N/A	74 ± 6.1	53.8%	N/A	N/A
FTLD-non-C9 (Frontal cortex)	n=30	60 ± 8.5	70 ± 7.6	59.3%	FTLD	*GRN* (n=8) none (n=22)
FTLD-non-C9 (Cerebellum)	n=32	60 ± 8.5	70 ± 7.6	59.3%	FTLD	*GRN* (n=8) none (n=24)
FTLD-C9 (Frontal cortex)	n=15	57.7 ± 6.2	65.3 ± 7.0	60.0%	FTLD	*C9orf72* het (n=13) *C9orf72* homo (n=1) *C9orf72* het + *GRN* (n=1)
FTLD-C9 (Cerebellum)	n=15	57.7 ± 6.2	65.3 ± 7.0	60.0%	FTLD	*C9orf72* het (n=13) *C9orf72* homo (n=1) *C9orf72* het + *GRN* (n=1)

Supplementary Table 2. Genotypes of Drosophila stocks used.

Stock	Genotype
w1118	w[1118]
v-w+	w v^1
elavGS	w[1118]; P{elavGSGAL4}
GMR-GAL4	w[1118]; P{GMRGAL4}
UAS-$(G_4C_2)_{36}$	w[1118]; P{UAS-GGGGCC.36}attP40
UAS-$(GR)_{36}$	w[1118]; P{UAS-GR.36}attP40
RNA-only	w[1118]; P{UAS-GGGGCC.36RO}attP40
UAS-FASN1	w[1118]; P{UAS-FASN1.G}
UAS-FASN2	w[1118]; P{UAS-FASN2.G}
UAS-FASN1 RNAi	w[1118]; P{GD14739}2
UAS-Desat1	w[1118]; P{w[+mC]=UAS-Desat1.S}16
UAS-Desat1[42]	w[1118]; P{UAS-Desat1[42]}3
UAS-Desat1 RNAi	v^1 w[1118]; P{TRiP.HMS01654)attP40
UAS-FAT-2	w[1118]; P{w[+mC]=UAS-Cefat-2.S}3

Supplementary Table 3. Primer sequences

Primer name	Sequence
AcCoAS_for:	GAGCCACTTCAGTGATTTTCG
AcCoAS_rev:	ACTTCATGAGGGCACGAATC
FASN1_for:	GCTTGCTCCAGTTCTCTGTA
FASN1_rev:	GTATCCATTGCCAGACTCAT
Desat1_for:	CCGGAGTGCTCTTCGAGTG
Desat1_rev:	CAGCCAGATGGAGGTAACCG
Tubulin_for:	TGGGCCCGTCTGGACCACAA
Tubulin_rev:	TCGCCGTCACCGGAGTCCAT

Supplementary Table 4. i^3iPSC line information

iPSC line name	Common name	Sex (M/F)	Clinical diagnoses	Number of G_4C_2 repeats	Source of iPSCs
BS6	C9orf72 line 1	F	ALS/FTD	~750	Chandran lab, Univ. of Edinburgh
BS6-2H9 (Isogenic control of BS6)	Control line 1	F	N/A	Repeats removed by CRISPR-Cas9	Chandran lab, Univ. of Edinburgh
WTC11	Control line 2	M	N/A	N/A	Commercial
KOLF2.1J-D08	C9orf72 line 2	M	N/A	KOLF2.1J with ~ 224 repeats knocked-in	iNDI (*84, 85*)
KOLF2.1J-F05	C9orf72 line 3	M	N/A	KOLF2.1J with ~ 202 repeats knocked-in	iNDI (*84, 85*)
KOLF2.1J	Control line 3	M	N/A	Parental line	iNDI (*84, 85*)

Supplementary Table 5. Demographic Information for iPSN lines from Cedars Sinai

iPSC line name	Age at collection	Sex (M/F)	Clinical diagnosis
CS0201	56	F	Non-neurologic control
CS0002	51	M	Non-neurologic control
CS0206	72	F	Non-neurologic control
CS9XH7	53	M	Non-neurologic control
CS8PAA	58	F	Non-neurologic control
CS1ATZ	60	M	Non-neurologic control
CS2AE8	50	F	Non-neurologic control
CS3FGT	64	M	Non-neurologic control
CS0NKC	52	F	C9orf72
CS0LPK	67	M	C9orf72
CS0BUU	63	F	C9orf72
CS7VCZ	64	M	C9orf72
CS6ZLD	Unknown	F	C9orf72
CS8KT3	60	M	C9orf72
CS3MG8	52	F	TDP-43 M337V
CS5ZLD	62	F	TDP-43 N390D
CS8EDM	63	F	TDP-43 G384R
CS2BFU	44	M	SOD1 A5V
CS2RJV	57	M	SOD1 G94A
CS7AF6	63	M	SOD1 I114T
