# Supplementary material for: Neuronal polyunsaturated fatty acids are protective in ALS/FTD
Source: Nat Neurosci. 2025 Feb 25;28(4):737–47. doi: 10.1038/s41593-025-01889-3 (PMC11976277; doi:10.1038/s41593-025-01889-3)
Supplement: Supplementary file 2 — Reporting Summary [file 41593_2025_1889_MOESM2_ESM.pdf]

nature portfolio

Corresponding author(s): Adrian Isaacs

Last updated by author(s): Dec 2, 2024

Reporting Summary

Nature Portfolio wishes to improve the reproducibility of the work that we publish. This form provides structure for consistency and transparency in reporting. For further information on Nature Portfolio policies, see our Editorial Policies and the Editorial Policy Checklist.

Statistics

For all statistical analyses, confirm that the following items are present in the figure legend, table legend, main text, or Methods section.

n/a	Confirmed	
☐	☒	The exact sample size (*n*) for each experimental group/condition, given as a discrete number and unit of measurement
☐	☒	A statement on whether measurements were taken from distinct samples or whether the same sample was measured repeatedly
☐	☒	The statistical test(s) used AND whether they are one- or two-sided<br>*Only common tests should be described solely by name; describe more complex techniques in the Methods section.*
☒	☐	A description of all covariates tested
☐	☒	A description of any assumptions or corrections, such as tests of normality and adjustment for multiple comparisons
☐	☒	A full description of the statistical parameters including central tendency (e.g. means) or other basic estimates (e.g. regression coefficient) AND variation (e.g. standard deviation) or associated estimates of uncertainty (e.g. confidence intervals)
☐	☒	For null hypothesis testing, the test statistic (e.g. *F*, *t*, *r*) with confidence intervals, effect sizes, degrees of freedom and *P* value noted<br>*Give P values as exact values whenever suitable.*
☒	☐	For Bayesian analysis, information on the choice of priors and Markov chain Monte Carlo settings
☒	☐	For hierarchical and complex designs, identification of the appropriate level for tests and full reporting of outcomes
☒	☐	Estimates of effect sizes (e.g. Cohen's *d*, Pearson's *r*), indicating how they were calculated

Our web collection on statistics for biologists contains articles on many of the points above.

Software and code

Policy information about availability of computer code

Data collection	No custom code used.
Data analysis	GraphPad Prism version 10.0.2, ImageJ 1.53

For manuscripts utilizing custom algorithms or software that are central to the research but not yet described in published literature, software must be made available to editors and reviewers. We strongly encourage code deposition in a community repository (e.g. GitHub). See the Nature Portfolio guidelines for submitting code & software for further information.

Data

Policy information about availability of data

All manuscripts must include a data availability statement. This statement should provide the following information, where applicable:

- Accession codes, unique identifiers, or web links for publicly available datasets
- A description of any restrictions on data availability
- For clinical datasets or third party data, please ensure that the statement adheres to our policy

All transcriptomic data generated in this study is deposited in GEO, accession GSE255099. All lipidomic data generated from Drosophila brains, i3Neurons and post-mortem brains are publicly available for user-friendly exploration in the recently described NeuroLipidAtlas (https://neurolipidatlas.nl/) and can be found by selecting “Isaacs” as the contributing lab. Source data for all figures has been provided with this manuscript.

Research involving human participants, their data, or biological material

Policy information about studies with human participants or human data. See also policy information about sex, gender (identity/presentation), and sexual orientation and race, ethnicity and racism.

Reporting on sex and gender	Sex is indicated for iPSC lines and post-mortem brains
Reporting on race, ethnicity, or other socially relevant groupings	NA
Population characteristics	Age range 45-89, mean 69.7.
Recruitment	NA
Ethics oversight	Ethical approval was received from the NHS Health Research Authority East of England - Essex Research Ethics Committee (REC reference 18/EE/0293). All samples were collected with informed consent.

Note that full information on the approval of the study protocol must also be provided in the manuscript.

Field-specific reporting

Please select the one below that is the best fit for your research. If you are not sure, read the appropriate sections before making your selection.

☒ Life sciences ☐ Behavioural & social sciences ☐ Ecological, evolutionary & environmental sciences

For a reference copy of the document with all sections, see nature.com/documents/nr-reporting-summary-flat.pdf

Life sciences study design

All studies must disclose on these points even when the disclosure is negative.

Sample size	We did not perform sample size calculations. Sample sizes were based on our own, or literature, experience of the number of samples required to observe biologically meaningful differences between groups.
Data exclusions	None
Replication	We replicated lipidomics findings in iPSC -neurons by running n=3 inductions for each C9orf72 line and further confirmed using ASO treatments to abrogate changes. All experiments were successfully replicated with biological replicates as shown in the figures.
Randomization	Groups were assigned by their genetic status so did not require randomisation
Blinding	Blinding was not used where we used non-subjective measurements: death, lipid levels, DPR levels, transcript levels; but was performed for Drosophila feeding assays.

Reporting for specific materials, systems and methods

We require information from authors about some types of materials, experimental systems and methods used in many studies. Here, indicate whether each material, system or method listed is relevant to your study. If you are not sure if a list item applies to your research, read the appropriate section before selecting a response.

Materials & experimental systems

n/a	Involved in the study	
☐	☒	Antibodies
☐	☒	Eukaryotic cell lines
☒	☐	Palaeontology and archaeology
☐	☒	Animals and other organisms
☒	☐	Clinical data
☒	☐	Dual use research of concern
☒	☐	Plants

Methods

n/a	Involved in the study	
☒	☐	ChIP-seq
☒	☐	Flow cytometry
☒	☐	MRI-based neuroimaging

Antibodies

Antibodies used	Anti-poly(GP) 658, custom-made by Eurogentec; anti-poly(GA) clone 5E9, MABN889, Merck Millipore; anti-poly(GA) clone 5F2, kind gift of Prof D. Edbauer.
Validation	Anti-poly(GP) 658 and anti-poly(GA) clone 5E9, MABN889, Merck Millipore were validated by us previously in Quaegebeur et al Acta Neuropathol Comms 8, 184 (2020). Anti-polyGA clones 5E9 and 5F2 were both validated in Mackenzie et al Acta Neuropathol 126: 859–879 (2013).

Eukaryotic cell lines

Policy information about cell lines and Sex and Gender in Research

Cell line source(s)	WTC11 human iPSC line was from the Coriell Institute. BS6, female and BS6-2H9, female, patient lines provided by Prof. Siddhartan Chandran. KOLF2.1J, male, KOLF2.1J-D08, male, KOLF2.1J-F05 male, lines provided by iNDI (Jackson Laboratories). Human iPSC lines (CS0201, CS0002, CS0206, CS9XH7, CS8PAA, CS1ATZ, CS0NKC, CS0LPK, CS0BUU, CS7VCZ, CS6ZLD, CS8KT3) from Cedars-Sinai used by Dr Alyssa N Coyne. HEK293T cells were from ATCC.
Authentication	Validation performed by AnswerALS, Jackson Laboratory or were previously published (Selvaraj et al 2018). HEK293T and WTC11 cells were not re-authenticated in our laboratory.
Mycoplasma contamination	We confirmed the lines were mycoplasma negative over the course of the experiments as we test regularly (monthly).
Commonly misidentified lines (See ICLAC register)	No commonly misidentified cell lines used.

Animals and other research organisms

Policy information about studies involving animals; ARRIVE guidelines recommended for reporting animal research, and Sex and Gender in Research

Laboratory animals	Drosophila Melanogaster strain w1118 or v-w1118, used throughout lifespan
Wild animals	NA
Reporting on sex	All experiments were performed on female flies
Field-collected samples	NA
Ethics oversight	Ethical approval not required for work on Drosophila Melanogaster

Note that full information on the approval of the study protocol must also be provided in the manuscript.

Plants

Seed stocks	*Report on the source of all seed stocks or other plant material used. If applicable, state the seed stock centre and catalogue number. If plant specimens were collected from the field, describe the collection location, date and sampling procedures.*
Novel plant genotypes	*Describe the methods by which all novel plant genotypes were produced. This includes those generated by transgenic approaches, gene editing, chemical/radiation-based mutagenesis and hybridization. For transgenic lines, describe the transformation method, the number of independent lines analyzed and the generation upon which experiments were performed. For gene-edited lines, describe the editor used, the endogenous sequence targeted for editing, the targeting guide RNA sequence (if applicable) and how the editor was applied.*
Authentication	*Describe any authentication procedures for each seed stock used or novel genotype generated. Describe any experiments used to assess the effect of a mutation and, where applicable, how potential secondary effects (e.g. second site T-DNA insertions, mosiacism, off-target gene editing) were examined.*
